# Low CCR5 expression protects HIV-specific CD4+ T cells of elite controllers from viral entry

**DOI:** 10.1038/s41467-022-28130-0

**Published:** 2022-01-26

**Authors:** Mathieu Claireaux, Rémy Robinot, Jérôme Kervevan, Mandar Patgaonkar, Isabelle Staropoli, Anne Brelot, Alexandre Nouël, Stacy Gellenoncourt, Xian Tang, Mélanie Héry, Stevenn Volant, Emeline Perthame, Véronique Avettand-Fenoël, Julian Buchrieser, Thomas Cokelaer, Christiane Bouchier, Laurence Ma, Faroudy Boufassa, Samia Hendou, Valentina Libri, Milena Hasan, David Zucman, Pierre de Truchis, Olivier Schwartz, Olivier Lambotte, Lisa A. Chakrabarti

**Affiliations:** 1 Virus and Immunity Unit, Institut Pasteur, Université de Paris, Paris, France; 2 CNRS UMR3569, Paris, France; 3 Bioinformatics and Biostatistics Hub, Department of Computational Biology, Institut Pasteur, Université de Paris, Paris, France; 4 AP-HP Hôpital Necker-Enfants Malades, Laboratoire de Microbiologie clinique, Paris, France; 5 CNRS 8104, INSERM U1016, Université Paris Descartes, Sorbonne Paris Cité, Faculté de Médecine, Paris, France; 6 Biomics Platform, C2RT, Institut Pasteur, Université de Paris, Paris, France; 7 INSERM U1018, Center for Research in Epidemiology and Population Health (CESP), Le Kremlin-Bicêtre, France; 8 Cytometry and Biomarkers (CB UTechS), Translational Research Center, Institut Pasteur, Université de Paris, Paris, France; 9 HIV Unit, Foch Hospital, Suresnes, France; 10 AP-HP, Infectious and Tropical Diseases Department, Raymond Poincaré Hospital, Garches, France; 11 INSERM U1184, Université Paris Sud, CEA, Center for Immunology of Viral Infections and Autoimmune Diseases, Le Kremlin-Bicêtre, France; 12 AP-HP, Department of Internal Medicine and Clinical Immunology, University Hospital Paris Sud, Le Kremlin-Bicêtre, France

**Keywords:** Chemokines, Retrovirus, Viral membrane fusion, HIV infections

## Abstract

HIV elite controllers maintain a population of CD4 + T cells endowed with high avidity for Gag antigens and potent effector functions. How these HIV-specific cells avoid infection and depletion upon encounter with the virus remains incompletely understood. Ex vivo characterization of single Gag-specific CD4 + T cells reveals an advanced Th1 differentiation pattern in controllers, except for the CCR5 marker, which is downregulated compared to specific cells of treated patients. Accordingly, controller specific CD4 + T cells show decreased susceptibility to CCR5-dependent HIV entry. Two controllers carried biallelic mutations impairing CCR5 surface expression, indicating that in rare cases CCR5 downregulation can have a direct genetic cause. Increased expression of β-chemokine ligands upon high-avidity antigen/TCR interactions contributes to autocrine CCR5 downregulation in controllers without CCR5 mutations. These findings suggest that genetic and functional regulation of the primary HIV coreceptor CCR5 play a key role in promoting natural HIV control.

## Abstract (web summary)

Here, Claireaux et al. show that people who naturally control HIV infection express lower levels of the viral co-receptor CCR5 in specific CD4^+^ T cells, and that this results from mutations or receptor internalization by CD4^+^ T cell-produced chemokines.

## Introduction

The rare individuals who spontaneously control HIV replication in the absence of antiretroviral therapy have a very low risk of progression to AIDS^1,2^. These patients, called elite controllers or HIV controllers, represent fewer than 0.5% of HIV-1 infected individuals, but can maintain a healthy status and preserved CD4 + T cell counts for over decades^3^. Most of HIV controllers appear infected with replication-competent HIV strains, suggesting that host factors must play a role in ensuring viral control^4^. Genetic studies uncovered a robust association between certain MHC class I alleles and HIV control, consistent with the involvement of CD8 + T cells in containing HIV^5^. Indeed, HIV controllers harbor antiviral CD8 + T cells that are highly efficient at eliminating infected target cells in an MHC I restricted fashion^6,7^. MHC I genetics, however, does not fully account for HIV control, as the vast majority of infected patients carrying protective MHC I alleles still go on to develop progressive HIV infection. Further characterization of host factors underlying HIV control remains a priority, as this may help understand how the human immune system can resist AIDS, and may inform the development of vaccines and immunotherapies directed at HIV.

Converging evidence indicates that HIV-specific CD4 + T cell responses are also particularly efficient in HIV controllers. Antiviral CD4 + T cells are not only preserved in numbers, as expected from the lack of virus-induced depletion, but also show improved functions as compared to those of treated and viremic patients^8–10^. CD4 + T cells from controllers maintain high proliferative capacity in response to HIV antigens and show only limited expression of immune exhaustion markers^11^. Effector functions such as polyfunctional cytokine secretion and help provided to B cells appear superior in HIV controllers compared to other patients groups^12–14^. In addition, CD4 + T cell cytotoxic capacity in acute HIV infection associates with a lower viral load setpoint later on, suggesting that CD4 + T cells may also directly contribute to HIV control by eliminating infected cells^15^. A key factor underlying the potency of CD4 antiviral functions lies in the nature of the T cell receptors (TCRs) expressed by HIV-specific CD4 + T cells. We previously reported that Gag-specific CD4 + T cells of controllers expressed TCRs of higher affinities than those of treated patients, resulting in more sensitive detection of viral antigens^16^. Molecular characterization of TCRs specific for the most immunodominant Gag epitope, Gag293, uncovered the presence of shared (or public) high-affinity TCRs that were preferentially expressed in HIV controllers^17^. Importantly, the transfer of a public high-affinity TCR to healthy donor cells was sufficient to recapitulate the series of properties characteristic of controller CD4 + T cells, including high antigen sensitivity, polyfunctional cytokine secretion, and cytotoxic capacity^18^.

The mechanisms underlying the selection and persistence of high-affinity CD4 + T cells in HIV controllers remain poorly understood. It is not clear in particular how these cells escape infection and depletion by HIV, considering that high-affinity CD4 + T cells are exquisitely sensitive to Gag antigens, and are thus likely the first to become activated and productively infected upon virus encounter. The possibility that controller CD4 + T cells are intrinsically less infectable by HIV is supported by some but not all groups^19–22^ who examined the susceptibility of patient CD4 + T cells to in vitro infection or their capacity to undergo HIV reverse transcription and integration^23^. Genetic associations between HLA II alleles and HIV control have been reported, though these appear less robust than those observed MHC I^12,24^. In the absence of strong genetic associations, the factors responsible for the preservation of high-affinity CD4 + T cells in HIV controllers remain to be elucidated. To address this issue, we set to compare the differentiation and activation status of Gag293-specific CD4 + T cells obtained from HIV controllers and long-term treated patients. The use of MHC II tetramer technology enabled an ex vivo characterization of rare HIV-specific CD4 + T cells at the single cell level, without perturbations associated to in vitro activation.

We obtained evidence for a lower expression of CCR5 in HIV-specific CD4 + T cells of controllers, accounting for lower susceptibility to HIV entry. Decreased CCR5 expression had a genetic cause in two cases, and more broadly depended on increased expression of the β-chemokines CCL5/RANTES and CCL3/MIP-1α, which could drive CCR5 downregulation upon strong TCR stimulation. Therefore, genetic and TCR-dependent regulation of the CCR5 pathway provides a mechanistic explanation for the preservation of the specific CD4 + T cell population in HIV controller patients.

## Results

We set to analyze the surface phenotype and transcriptional profile of HIV-specific CD4 + T cells in HIV controllers with long-term viral suppression (HIC group, *n* = 12). Patients from the ANRS CO21 CODEX cohort were included based on efficient viral load suppression (<50 HIV-1 RNA copies/mL) for >5 years. The reference group consisted of treated patients (ART group, *n* = 15) who had a similarly long viral suppression (<50 HIV-1 RNA copies/mL for >5 years), ensuring that immunological differences between groups did not depend primarily on variations in HIV antigen availability. Ultrasensitive viral load analysis showed a viral load <10 copies/mL in 11 out of 12 HIV controllers (Supplementary Table 1), pointing to the efficiency of viral control in recruited patients.

### Distinct transcriptional profile in Gag293-specific CD4 + T cells

**Fig. 1:**
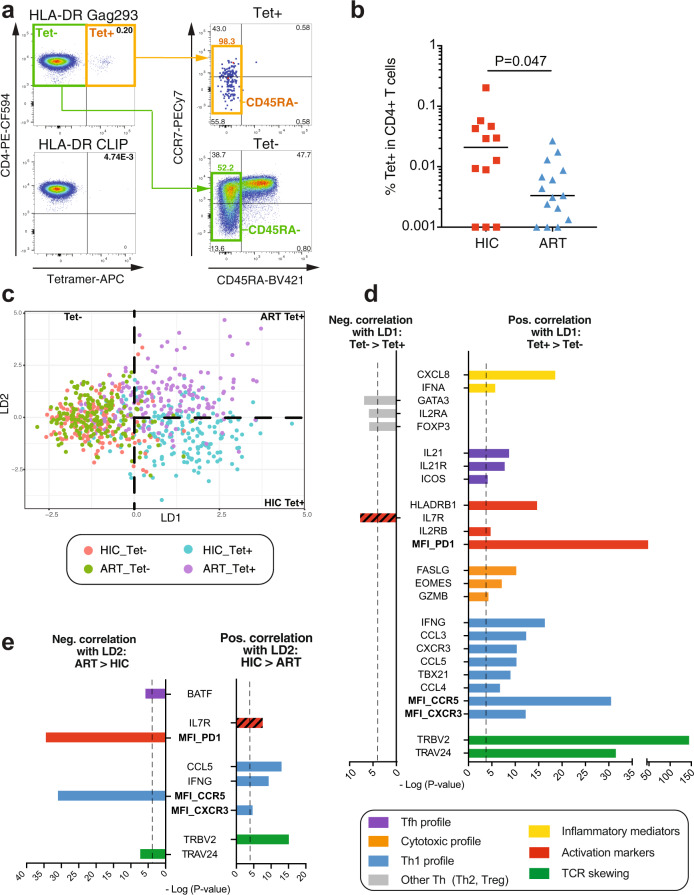
Dominant Th1 differentiation pattern in Gag293-specific cells of controllers. **a** Example of MHC II tetramer labeling in controller CD4 + T cells, with a Gag293-loaded tetramer (top left) compared to a CLIP-loaded control tetramer (bottom left). The frequency of CD45RA− memory cells is increased in the tetramer-positive (Tet+) population (right). **b** Percentage of Gag293-specific cells in CD4 + T cells of controllers (HIC, *n* = 12) and treated patients (ART, *n* = 15). Medians were compared with the Mann-Whitney test. **c** Distribution of *n* = 700 single CD4 + T cells by linear discriminant analysis (LDA). **d**, **e** List of variables that are most significantly correlated with the LD1 (**d**) or LD2 (**e**) discriminant directions defined in the LDA, with a threshold *P*-value < 10^−^^4^. *P* values were computed for the Pearson correlations, without corrections for multiple comparisons. Genes are reported in plain text while mean fluorescence intensities (MFI) of proteins are reported in bold. Variables are color-coded according to their implication in T cell differentiation pathways (e.g., Th1 variables in blue). The bar for IL7R is hatched because this marker decreases upon activation. Source data are provided as a Source Data file.

The study focused on CD4 + T cells specific for the most conserved epitope in HIV capsid, Gag293, as this epitope is remarkably immunodominant, with responses elicited in over 70% of HIV controllers and about half of the treated patients^16^. Gag293 can be presented by multiple HLA II alleles^17^, which enabled the recruitment of patients with expressing at least one of 7 HLA DRB1 alleles (Supplementary Table 1). Each PBMC sample was labeled in parallel with a Gag293-loaded MHCII tetramer and a control tetramer loaded with the CLIP peptide. Tetramer-positive (Tet+) CD4 + T cells showed a predominantly memory phenotype (Fig. 1a; gating shown in Supplementary Fig. 1a) and were detected at higher frequency in HIV controllers than in treated patients (Fig. 1b; median: HIC 0.021%, ART 0.0033%, *P* < 0.05), consistent with the notion of preserved HIV-specific CD4 responses in controlled HIV infection^14^.

The Tet+ and Tet− populations were analyzed at the single-cell level using flow cytometry-based index sorting combined with a multiplexed RT-qPCR assay (Biomark, Fluidigm). Using this approach, we measured the expression of 47 genes (Supplementary Table 2) and 6 surface proteins in 9 HIV controllers and 9 treated patients who showed a detectable Tet+ population during sorting (Supplementary Fig. 1a). The analysis was restricted to memory CD45RA− CD4 + T cells, to avoid a bias due to the very different proportions of naive cells in the Tet+ and Tet− populations^14^. The combined gene and protein expression dataset for 700 single cells was analyzed by linear discriminant analysis (LDA), to define the linear combination of features (transcripts and proteins) that best separated the 4 subgroups of interest (HIC Tet+, ART Tet+, HIC Tet−, and ART Tet−)^25^. As shown on Fig. 1c, the first linear discriminant direction (LD1) discriminated cells mainly on their Tet status, pointing to a distinct gene expression profile in Gag293-specific cells. The second direction (LD2) discriminated cells according to the group of patients, with group separation visible mainly among Tet+ cells. Thus, differences between the HIC and ART patient groups appeared to be driven by HIV-specific cells.

### Dominant Th1 differentiation pattern in Gag293-specific CD4 + T cells of controllers

Examining the top variables that correlated most significantly with LD1 (p < 10^−^^4^; Fig. 1d) indicated that Tet+ cells were primarily characterized by the expression of genes and proteins involved in Th1 differentiation, including *IFNG, CCL3, CCL4, CCL5, CXCR3, CCR5, and TBX21* (coding for T-bet). Markers of cytotoxic capacity (*FASL, EOMES, GZMB*) also predominated in Tet+ cells. In contrast, markers of Th2 differentiation (*GATA3*) and of Treg differentiation (*IL2RA, FOXP3*) were negatively associated with HIV-specificity. Markers of Tfh differentiation (*ICOS, IL-21R, IL-21*) were slightly enriched in the Tet+ population, indicating that a degree of plasticity persisted in Gag293-specific CD4 + T cells. HIV-specificity was also associated with a clear increase in markers of immune activation (high expression of *HLA-DRB* and PD-1; low expression of *IL7R*) and of inflammation (*CXCL8, IFNA*), suggesting that HIV antigen-driven activation persisted even in patients with well-controlled viral loads. Last, expression of the TCR variable genes *TRBV2* and *TRAV24* were markedly enriched in Gag293-specific cells, consistent with our previous findings^17,18^.

Variables correlated with LD2 mainly distinguished HIC and ART Tet+ cells (Fig. 1c and e). Based on this observation, HIC Tet+ cells showed a more significant association with Th1 markers (*CCL5*, *IFNG*, and CXCR3), with the exception of the CCR5 marker, which was more prominent in ART Tet+ group. Gag293-specific CD4 + T cells of treated patients were also characterized by high expression of the PD-1 protein, consistent with the notion of persisting immune activation and/or exhaustion in spite of antiretroviral therapy^2,14^. Analysis of genes communities that were coexpressed in Gag293-specific cells (Supplementary Fig. 1b) revealed a larger network of correlated genes in the specific cells of controllers. Interestingly, this network included key markers of cytotoxic function (*PRF1*, *GZMB*, and *EOMES*), pointing to the induction of a cytotoxic CD4 + T cell differentiation program in naturally controlled HIV infection.

**Fig. 2:**
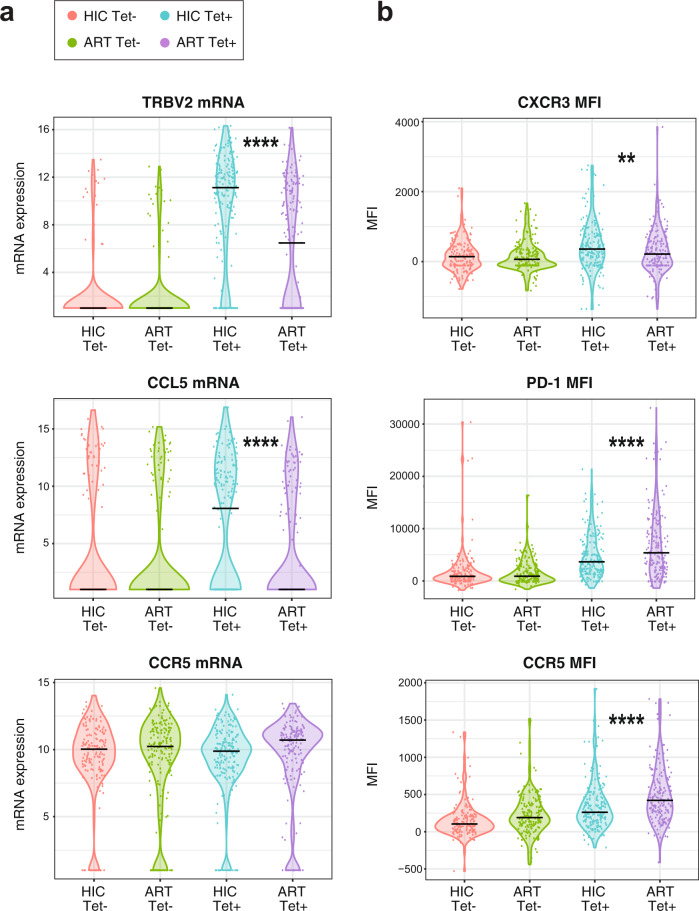
Gag293-specific cells of controllers are characterized by high CCL5 but low CCR5 expression. **a**, **b** Distribution of gene expression values (**a**) and protein MFI (**b**) for selected variables in the 4 single cell groups (HIC Tet+ *n* = 188, HIC Tet− *n* = 160, ART Tet+ *n* = 170, ART Tet− *n* = 182). Significant *P* values measured by a Mann-Whitney test between the HIC Tet+ and ART Tet+ groups are indicated by stars (**P < 0.01, ****P < 0.0001).

In a statistical analysis of individual variables^26^, two genes proved to be significantly more expressed in HIC than ART Tet+ cells after correcting for multiple comparisons: the chemokine *CCL5* (*p* < 0.0001), a major ligand of CCR5, and the variable TCR gene *TRBV2* (*P* < 0.0001) (Fig. 2a and Supplementary Fig. 2a). Of note, *CCR5* receptor transcripts did not significantly differ between the two populations of Tet+ cells (Fig. 2a), though a trend was noted for higher expression in ART Tet+ cells (*P* = 0.018 without correction for multiple comparisons). The two other main ligands of CCR5, the chemokines *CCL3* and *CCL4*, did not distinguish between the HIC and ART groups (Supplementary Fig. 3). None of the genes studied distinguished the non-specific Tet− cells from the HIC and ART groups (Supplementary Fig. 2). Regarding surface proteins, the analysis revealed a highly significant increase of PD-1 (*p* < 0.0001) expression in ART Tet+ compared to HIC Tet+ cells, suggesting that chronic antigenic stimulation and/or immune exhaustion persisted in the HIV-specific compartment of treated patients (Fig. 2b and Supplementary Fig. 2c). The CD8 marker, which can be viewed as an activation marker in CD4 + T cells, was also increased in ART Tet+ cells (*p* = 0.029), while the CXCR3 marker, indicative of Th1 differentiation, was increased in HIC Tet+ cells (*p* = 0.009). CCR5 protein expression was generally lower in Tet− than Tet+ cells (Fig. 2b) and showed a highly significant increase in ART Tet+ compared to HIC Tet+ cells (Supplementary Fig. 2c). Taken together, the single-cell analyses revealed a pattern of more advanced Th1 differentiation in Gag293-specific cells of controllers, with a potential for cytotoxic CD4 + T cell differentiation. The only Th1 marker that did not fit this pattern was CCR5, which reached higher levels in the specific cells of treated patients. Given the key role of CCR5 in mediating HIV entry^27^, we focused the study on an in-depth analysis of CCR5 expression and coreceptor function in HIV-specific CD4 + T cells.

### Low CCR5 expression associates with preservation of the HIV-specific CD4 + T cell population

**Fig. 3:**
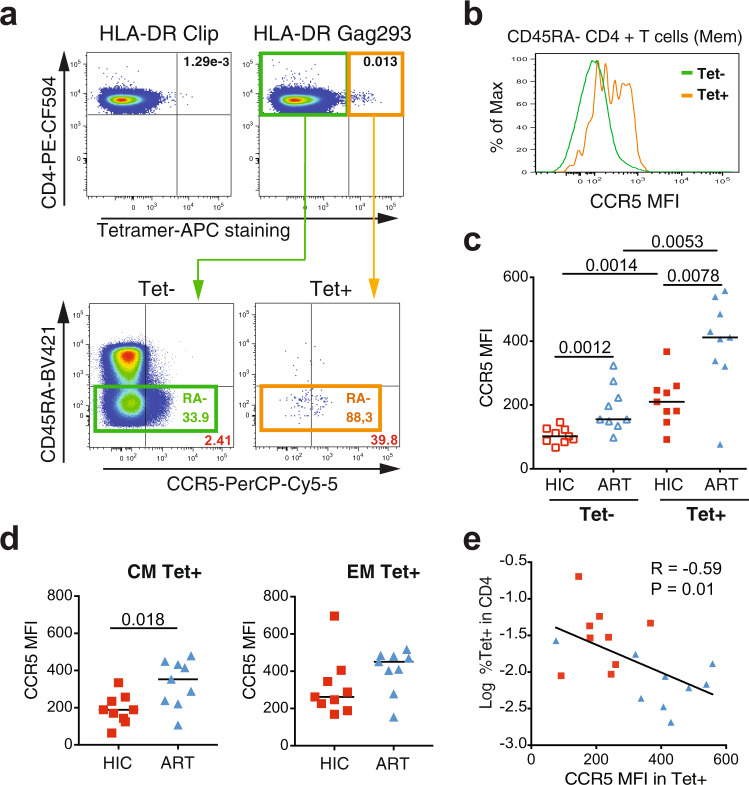
Low CCR5 expression correlates with the persistence of Gag293-specific CD4 + T cells. **a** Example of CCR5 staining in Gag293-specific (Tet+) and non-specific (Tet−) controller CD4 + T cells. **b** Comparison of the CCR5 MFI in the Tet+ and Tet− populations shown in **a**, after gating in the CD45RA− memory (Mem) population. **c** Comparison of CCR5 MFI in the Tet+ and Tet− populations of controller (HIC) and treated patient (ART) Mem CD4 + T cells. **d** Comparison of CCR5 expression in the central memory (CM) and effector memory (EM) subsets of Tet+ cells. Significant differences between medians computed with the Mann-Whitney test are reported in **c**–**d**. **e** Inverse correlation between the frequency of Gag293-specific Tet+ cells in CD4 + T cells and CCR5 expression in Mem Tet+ cells. The Spearman linear correlation coefficient R and the associated *P*-value are reported. HIC Tet+ red squares; ART Tet+ blue triangles.

We first analyzed CCR5 expression in bulk CD4 + T cell populations rather than at the single-cell level, to validate our analysis on a larger number of cells obtained from the same patients (Fig. 3a). We verified that the HEK/1/85a CCR5 antibody used throughout the study gave optimal discrimination of CCR5+ cells compared to other commonly used antibodies (Supplementary Fig. 4a). We confirmed the hierarchy observed at the single-cell level (Fig. 3b and c), with close to a doubling of CCR5 mean fluorescence intensity (MFI) between specific and non-specific cells and another doubling of the MFI between HIC Tet+ and ART Tet+ cells (medians: HIC Tet− 102; ART Tet− 155; HIC Tet+ 210; ART Tet+ 412). Longitudinal analyses of Tet+ cells carried out for two controllers showed that Tet+ cell frequencies were stable over a two-year period (Supplementary Fig. 5). Moreover, CCR5 expression levels in Tet+ cells also proved stable during this period, suggesting that low CCR5 expression could be maintained in the long-term in controller-specific CD4 + T cells. Measurement of CCR5 expression in the central memory (CM: CD45RA−  CCR7+) and effector memory (EM: CD45RA− CCR7−) specific CD4 + T cells subsets showed that the difference between the HIC and ART groups was more marked in the CM Tet+ compartment (*P* = 0.018), while the EM Tet+ compartment showed only a trend for higher CCR5 expression in the ART group (Fig. 3d). The frequencies of CM cells were equivalent in the Tet+ and Tet− subsets, both in the HIC and the ART groups (Supplementary Fig. 4b). Thus, the differences in CCR5 expression between the HIC and ART Tet+ or Tet− populations did not result from variations in proportions of the CM and EM subsets, but rather from differences in CCR5 expression within these subsets. Interestingly, CCR5 expression in Gag293-specific cells correlated inversely with the frequency of these specific cells (*R* = −0.59, *P* = 0.01; Fig. 3e), raising the possibility that low CCR5 expression protected HIV-specific CD4 + T cells from infection and depletion. A similar correlation was found when restricting the CCR5 analysis to the CM Tet+ subsets (*R* = −0.58, *P* = 0.01), but was not significant when analyzing the EM Tet+ subsets (*R* = −0.38, *P* = 0.12) (Supplementary Fig. 4c–d). Thus, low CCR5 expression in the CM compartment was associated with a preservation of HIV-specific CD4 + T cell responses.

### Low susceptibility of controller specific CD4 + T cells to HIV entry

**Fig. 4:**
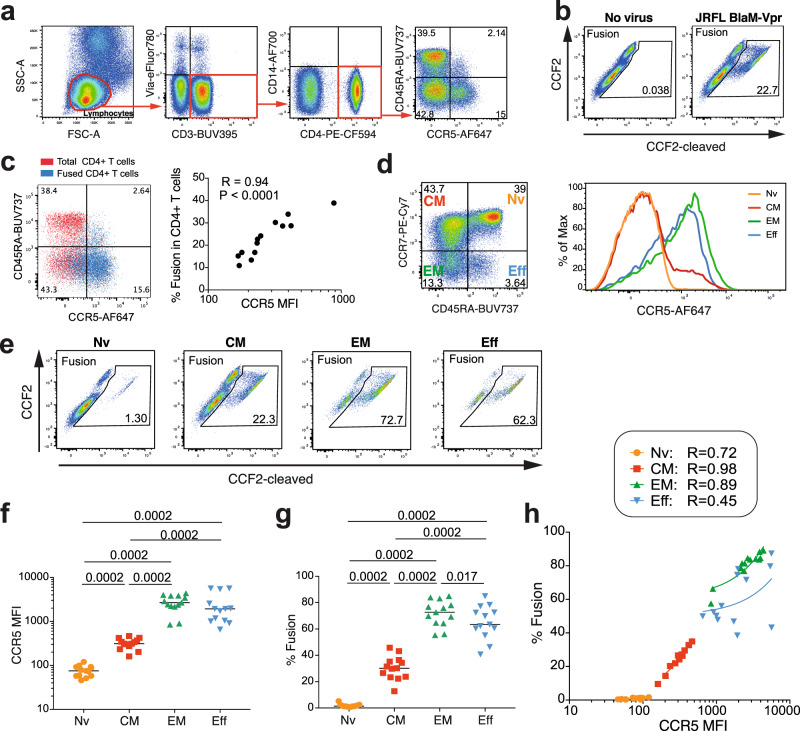
R5 HIV entry is strongly dependent on CCR5 expression. Fusion with the R5 HIV-1 reporter virus JR-FL BlaM-Vpr was analyzed in unstimulated primary CD4 + T cells from healthy donors (HD, *n* = 13). **a** Gating strategy for the analysis of HIV-1 fusion in CD4 + T cells. **b** Example of HIV-1 fusion in HD CD4 + T cells, as measured by the cleavage of the fluorescent FRET substrate CCF2. **c** Overlay plot illustrating that HIV-1 JR-FL fusion occurs predominantly in CCR5 + cells (left) and association between CCR5 expression (MFI) and fusion in HD CD4 + T cells (right). The Pearson correlation coefficient R is reported. **d** CCR5 expression in CD4 + T cell subsets from one HD. Naive (Nv), central memory (CM), effector memory (EM), and effector (Eff) subsets defined by CD45RA and CCR7 expression (left) show different CCR5 expression levels (right). **e** Example of HIV-1 JR-FL fusion in the 4 CD4 + T cell subsets. **f**, **g** Quantitation of CCR5 expression (**f**) and HIV-1 JR-FL fusion (**g**) in the 4 CD4 + T cell subsets. Significant differences between medians were computed with the Mann-Whitney test. **h** Correlations between CCR5 expression and HIV-1 JR-FL fusion in the 4 CD4 + T cell subsets. Pearson linear correlation coefficients are reported. Nv Orange circles, CM Red squares, EM Upward green triangles, Eff Downward blue triangles.

To evaluate the functional consequences of decreased CCR5 expression, we optimized a fusion assay designed to measure HIV entry in primary cells^28^. CCR5-using (R5) HIV-1 JR-FL virions carrying a Vpr-βlactamase reporter protein yielded fusion levels consistently above 10% in healthy donor resting CD4 + T cells, as measured by the cleavage of the βlactamase substrate CCF2-AM in target cells (Fig. 4a–c). Analysis of HIV fusion in healthy donor cells (HD, *n* = 13) revealed a tight correlation between the level of fusion in unstimulated CD4 + T cells and CCR5 expression (*R* = 0.94, *P* < 0.0001; Fig. 4c). The naive (Nv), CM, EM, and effector (Eff) CD4 + T cell subsets of HD differed markedly in CCR5 expression (Nv << CM < EM ≈ Eff−; Fig. 4d, f) and differed accordingly in their susceptibility to HIV entry (Fig. 4e, g). The correlation between fusion and CCR5 expression proved tighter in the CM (*R* = 0.98) than in the EM subset (*R* = 0.89) (Fig. 4h), indicating a stronger coreceptor dependency at intermediate CCR5 expression levels.

**Fig. 5:**
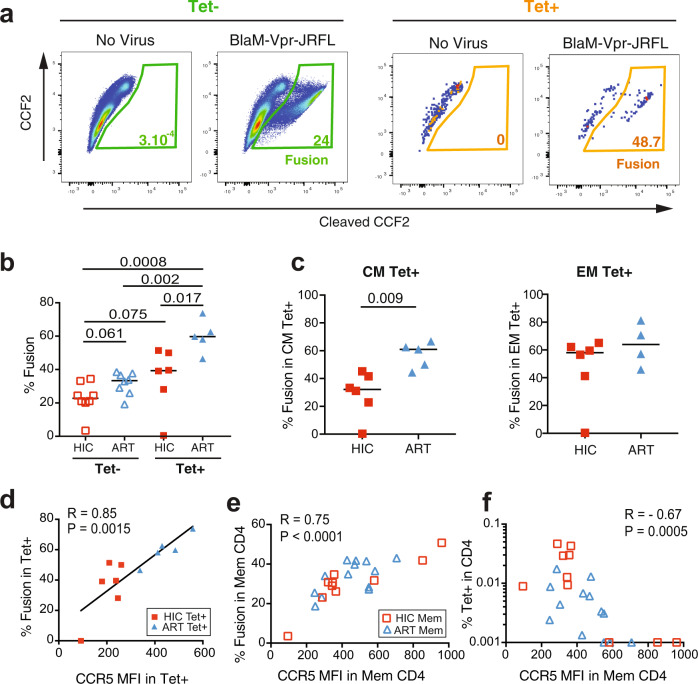
Low susceptibility of controller Gag293-specific CD4 + T cells to R5 HIV entry. HIV-1 JR-FL fusion was analyzed in Gag293-specific (Tet+) and non-specific (Tet−) memory CD4 + T cells from controllers (HIC) and treated patients (ART). **a** Example showing increased HIV fusion in Tet+ compared with Tet− cells from one controller. **b** Comparison of HIV fusion in Tet+ and Tet− cells from the HIC and ART groups. **c** Comparison of HIV fusion in central memory (CM, left) and effector memory (EM, right) Tet+ populations between the HIC and ART groups. **b**, **c** Differences between medians were computed with the Mann-Whitney test. **d** Positive correlation between CCR5 expression and fusion in the Tet+ population. **e** Positive correlation between CCR5 expression and fusion in the Mem (CD45RA− CD4 +) population. **f** Inverse correlation between Tet+ cells in CD4 + T cells and CCR5 expression in Mem cells. **d**–**f** Spearman correlation coefficients and associated P values are reported in plots. HIC Tet+ Filled red squares, ART Tet+ Filled blue triangles, HIC Mem Open red squares, ART Mem Open blue triangles.

We next applied the fusion assay to patient PBMC, to evaluate susceptibility to HIV entry in both the Tet+ and Tet− CD4 + T cell populations (Fig. 5a). Due to the scarcity of Tet+ cells, analysis of fusion in the Tet+ population was limited to samples with ≥2 × 10^7^ available PBMC. Fusion in the Tet− populations did not significantly differ between the HIC and ART groups, though a trend for higher fusion was noted in the latter group (Fig. 5b). In contrast, fusion was generally increased in Tet+ cells, and proved significantly higher in ART Tet+ than in HIC Tet+ cells (Fig. 5b; median HIC 39.3% vs. ART 59.7%, *P* = 0.017). When analyzing HIV entry in subsets of Tet+ cells, a highly significant difference was observed in the percentage of fused CM Tet+ cells between the HIC and ART groups (median HIC 32.2% vs. ART 61.0%, *P* = 0.009), while again the EM Tet+ cells showed no significant differences between groups (Fig. 5c). Susceptibility to HIV fusion in Gag293-specific cells strongly correlated with CCR5 expression (Fig. 5d; *R* = 0.85; *P* = 0.0015), providing a possible mechanistic explanation for the preservation of these cells in controlled HIV infection. A similar correlation was observed when the analysis was restricted to the CM but not to the EM subset of specific cells (Supplementary Fig. 4e, f), suggesting that low CCR5 expression was particularly relevant to the protection of the central memory compartment.

We next examined HIV entry in the total CD45RA− CD4 + T cell (Mem) compartment (Supplementary Fig. 4g). This analysis included HD (*n* = 13) as well as patients who had low or undetectable Tet+ populations, and who could not be included in previous analyses of tetramer-sorted cells. There were no significant differences in HIV-1 JR-FL fusion between Mem cells of the HIC and ART groups, while HIC Mem cells showed less efficient fusion than those of HD (*P* = 0.036). We also verified that susceptibility to a CXCR4-using virus, HIV-1 NL4-3, did not differ between groups (Supplementary Fig. 4h). The extent of JR-FL fusion correlated well with CCR5 expression in HIC and ART Mem cells (Fig. 5e; *R* = 0.75; *P* < 0.0001), confirming that CCR5 was a key determinant of susceptibility to HIV entry in a broad population of memory CD4 + T cells. Of note, several of the controller samples included in this analysis showed high CCR5 expression in Mem cells (Fig. 5e), contrasting with the initial analyses in the Tet− compartment (Fig. 3c). To clarify this point, we examined the frequency of Tet+ cells in function of CCR5 expression in Mem cells and observed a negative correlation between the two parameters (Fig. 5f; *R* = −0.67; *P* = 0.0005). The subset of controllers who showed high CCR5 expression in Mem cells had very low to undetectable frequencies of Gag293-specific cells, explaining why they were not included in the initial tetramer study. We conclude that low CCR5 expression is a characteristic of memory CD4 + T cells in some but not all HIV controllers and that mechanisms underlying HIV control are likely to be diverse. Importantly, only the group of controllers with low CCR5 expression showed a preserved Gag293-specific CD4 + T cell pool, suggesting that decreased CCR5 expression played a direct role in maintaining an efficient antiviral CD4 + T cell response.

### Biallelic CCR5 mutations in a controller with particularly low susceptibility to HIV entry

**Fig. 6:**
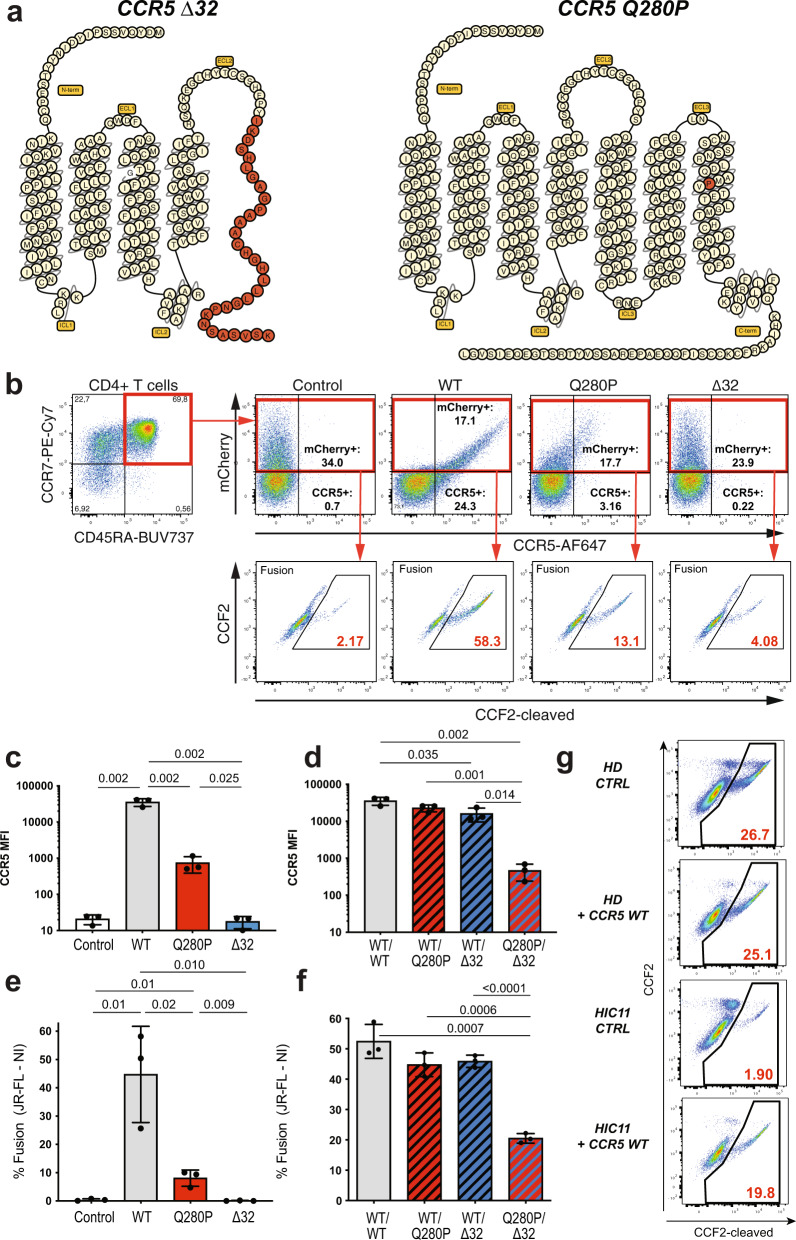
Biallelic mutations limit CCR5 surface expression in controller patient HIC11. **a** Schematic representation of CCR5 mutations Δ32 and Q280P found in patient HIC11. Modified amino acids are represented in red. ECL Extracellular loop. ICL Intracellular loop. **b**–**f** Effect of CCR5 mutations in primary CD4 + T cells: CCR5-T2A-mCherry wild-type (WT) or mutant plasmids were nucleofected in PBMC, and CCR5 expression was measured in the naive CD45RA + CCR7 + population. **b** Gating strategy for the combined analysis of CCR5 expression and HIV-1 JR-FL fusion. Nucleofection is measured by the % mCherry+ cells in naive CD4 + T cells. Fusion is measured by the % CCF2-cleaved+ cells in mCherry+ naive cells. **c**, **d** Quantification of CCR5 expression and (**e**, **f**) of HIV-1 JR-FL fusion, after single nucleofection (**c**, **e**) or co-nucleofection (**d**, **f**) of CCR5 plasmids. Means ± SD for *n* = 3 experiments are reported, with P values computed by Student’s t-test. **g** HIV-1 JR-FL fusion (red) is analyzed in total CD4 + T cells of one HD and of patient HIC11, either untreated (CTRL) or nucleoporated with CCR5 WT plasmid (*n* = 1 experiment).

Susceptibility to HIV entry showed individual variability in the HIC group, with one controller, in particular, having CD4 + T cells that were almost entirely resistant to HIV-1 JR-FL entry. Indeed, patient HIC11 showed only 3.4% fusion in Tet− cells and no detectable fusion in Tet+ cells (Fig. 5b). To search for CCR5 inactivating mutations, we sequenced the whole CCR5 locus (9 kb). Patient HIC11 proved to be heterozygous for the CCR5Δ32 mutation (nt. 551–582), a well-characterized 32 bp deletion found at an allelic frequency of approximately 10% in Caucasian individuals^27^. The Δ32 mutation generates a truncated CCR5 protein missing the last three transmembrane domains (TM5 to TM7), resulting in intracellular retention of the mutated protein (Fig. 6a, left). Interestingly, patient HIC11 carried an additional missense mutation at nt. 839 (A− > C) on the second CCR5 allele, which changed a glutamine to a proline residue at position 280 in TM7 (Fig. 6a, right). This Q280P mutation was rare, with a reported allelic frequency of 2.4 × 10^−5^ in the ExAC database.

None of the other controllers initially studied carried the CCR5Δ32 mutation. A heterozygous A335V mutation was detected in the CCR5 ORF of patients HIC07 and HIC10, the mutation consisting of a conservative alanine to valine substitution in the cytoplasmic domain (Table 1). This mutation is relatively frequent in individuals of African origin (up to 7% in the South-African population) and has not been shown to induce major changes in CCR5 expression or function^29,30^. Analyses of CCR5 promoter polymorphisms in the HIC group showed diverse haplotypes combinations (Table 1), which were previously associated with high (HHE/HHE), intermediate (HHC/HHE), or low (HHA/HHD) CCR5 expression^31^. Thus, neither the promoter nor the ORF analyses pointed to a common genetic factor that could account for low CCR5 expression in the entire controller group.

**Table 1:** Sequence analysis of the CCR5 locus in HIV controllers.

CCR5 reference haplotypes (a)	−2733	−2554	−2459	−2135	−2132	−2086	−1835	D32 551-582		
HHA	A	G	G	T	C	A	C	-		
HHB	A	**T**	G	T	C	A	C	-		
HHC	A	**T**	G	T	C	**G**	C	-		
HHD	A	**T**	G	T	**T**	A	C	-		
***HHE***	A	G	**A**	**C**	C	A	C	-		
***HHF***	A	G	**A**	**C**	C	A	**T**	-		
HHG*1	**G**	G	**A**	**C**	C	A	C	-		
HHG*2	**G**	G	**A**	**C**	C	A	C	D32		

HIV controller CCR5 genotype (b)	−2733	−2554	−2459	−2135	−2132	−2086	−1835	D32 551-582	other ORF mutations	CCR5 promoter haplotypes
HIC01	AA	GG	**AA**	**CC**	CC	AA	**TT**	-	-	***HHF/HHF***
HIC02	AA	**GT**	**AG**	**CT**	CC	**AG**	**CT**	-	-	***HHC/HHF***
HIC03	AA	**TT**	GG	TT	CC	**GG**	CC	-	-	HHC/HHC
HIC04	AA	**GT**	GG	TT	**CT**	AA	CC	-	-	HHA/HHD
HIC05	AA	GG	**AA**	**CC**	CC	AA	CC	-	-	***HHE/HHE***
HIC06	AA	GG	**AA**	**CC**	CC	AA	CC	-	-	***HHE/HHE***
HIC07	AA	**GT**	**AG**	**CT**	**CT**	AA	CC	-	A335V	***HHD/HHE***
HIC08	AA	**TT**	GG	TT	CC	**GG**	CC	-	-	HHC/HHC
HIC09	AA	**GT**	**AG**	**CT**	CC	**AG**	CC	-	-	***HHC/HHE***
HIC10	AA	GG	**AA**	**CC**	CC	AA	**CT**	-	A335V	***HHE/HHF***
HIC11	**AG**	GG	**AA**	**CC**	CC	AA	**CT**	D32	Q280P	HHF/HHG*2
HIC12	AA	**GT**	**AG**	**CT**	CC	**AG**	**CT**	-	-	***HHC/HHF***

HIV controller CCR5 genotype (c)	−2733	−2554	−2459	−2135	−2132	−2086	−1835	D32 551-582	other ORF mutations	CCR5 promoter haplotypes
HIC-D32-1	AG	GT	AG	CT	CC	AG	CC	D32	-	HHC/HHG*2
HIC-D32-2	AG	GT	AG	CT	CC	AG	CC	D32	F118L	HHC/HHG*2
HIC-D32-3	AG	GT	AG	CT	CC	AG	CC	D32	-	HHC/HHG*2

(a) List of CCR5 promoter haplotypes as defined by Mummidi et al. (J Biol Chem 275:18946, 2000).

Nucleotide changes compared to the reference haplotype HHA are in bold.

The names of deleterious haplotypes associated with high CCR5 expression are in bold italics.

(b) The whole CCR5 locus (9 kb) was sequenced by PacBio long-read technology for the 12 HIV controllers included in the MHCII tetramer study, except for HIC09, for whom the CCR5 locus was sequenced by the Sanger method except for HIC09, for whom the CCR5 locus was sequenced by the Sanger method.

CCR5 promoter haplotypes were imputed according to Mummidi et al.

Genotypes with at least one deleterious CCR5 promoter haplotype are in bold italics.

(c) Analysis of the CCR5 locus in 3 HIV controllers carrying the D32 mutation and showing low CCR5 expression.

### The Q280P mutation impairs CCR5 surface expression in primary CD4 + T cells

To analyze the functional consequences of the Q280P mutation in primary cells, plasmids expressing the wild type and mutated version of CCR5 along with mCherry were nucleoporated in unstimulated PBMC. Focusing the analysis on the naive CD4 + T cell subset, which barely expresses CCR5, allowed monitoring the expression of exogenously added CCR5 without interference from endogenous CCR5 (Fig. 6b). For equivalent nucleofection efficiency in primary CD4 + T cells, as shown by comparable mCherry expression levels, the Q280P mutant showed an impaired surface expression, with a 48x lower MFI compared to CCR5 WT (Fig. 6b, c, *P* = 0.002). However, expression of the Q280P mutant remained detectable at the surface of primary CD4 + T cells, in contrast to the lack of expression observed for the CCR5 Δ32 mutant (Fig. 6b, c). Accordingly, the fusion of HIV-1 JR-FL virus was significantly decreased in naive CD4 + T cells expressing the Q280 mutant (Fig. 6b, e, *P* = 0.02), but remained detectable, in contrast to the lack of fusion observed for cells expressing the Δ32 mutant. Further analyses of a FLAG-tagged CCR5 revealed an increased intracellular accumulation of the Q280P mutant, likely accounting for its decreased surface expression (Supplementary Fig. 6a). Analysis of mutant combinations showed that co-expression of the CCR5 Q280P and Δ32 mutants resulted in markedly decreased CCR5 surface expression (76x lower MFI compared to WT) and fusion in primary CD4 + T cells, thus recapitulating the phenotype observed in patient HIC11 (Fig. 6d, f). A similar decrease in CCR5 surface expression (61x lower MFI) was observed upon nucleoporation of the Q280P/Δ32 combination in CCR5 KO CD4 + T cells obtained by CRISPR technology (Supplementary Fig. 6b−d), confirming that the bi-allelic mutations identified in patient HIC11 profoundly impaired CCR5 surface expression. Nucleoporation of CCR5 WT in patient HIC11 PBMC proved sufficient to rescue JR-FL fusion (Fig. 6g and Supplementary Fig. 6e), indicating that the CCR5 mutations played a causal role in the low susceptibility to R5 HIV entry.

We then asked whether other controllers carrying the Δ32 mutation had additional CCR5 missense mutations on the second CCR5 allele. Out of 12 Δ32-positive controllers identified in the CODEX cohort, 3 showed low CCR5 surface expression, and of these 3, one carried an additional F118L mutation (Table 1). When expressed in primary CD4 + T cells, the F118L mutation resulted in a 29-fold decrease in CCR5 expression (Supplementary Fig. 6f, g), resulting in a phenotype similar to that observed for Q280P.

### Gag stimulation induces CCR5 downregulation via β-chemokine production

**Fig. 7:**
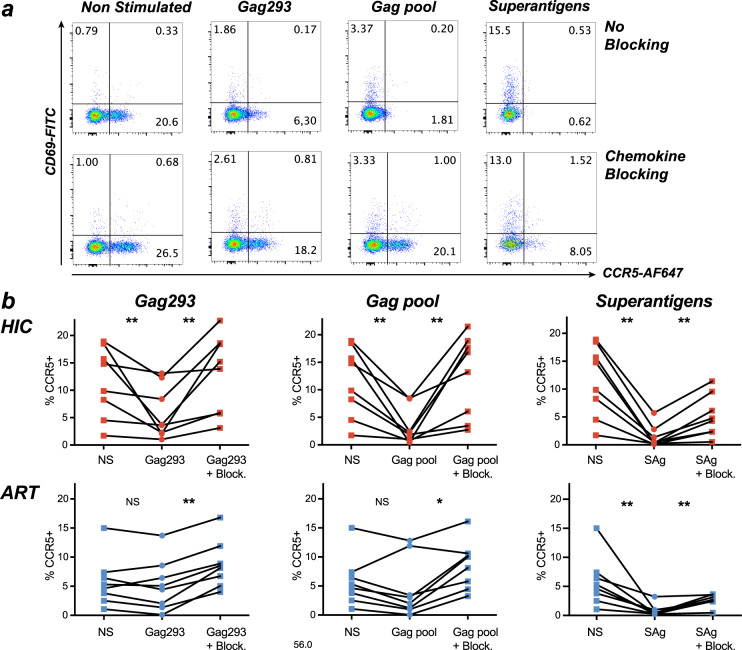
Downregulation of CCR5 via β-chemokine production upon antigenic stimulation. **a**, **b** Analysis of CCR5 expression in patient memory CD4 + T cells stimulated with Gag peptides or superantigens. **a** Example shown for one controller: CCR5 and CD69 expression were measured in CD45RA− CD4 + T cells after a 3-day stimulation of CD8-depleted PBMC with Gag293, a Gag peptide pool, or superantigens, in the absence (top) or presence (bottom) of β-chemokine blocking antibodies. **b** Quantitation of CCR5 expression in memory CD4 + T cells of HIC (top) and ART (bottom) without stimulation (NS) or post-stimulation, in the absence or presence (+Block.) of blocking β-chemokine antibodies. Variations in % CCR5 + cells were analyzed with the Wilcoxon matched-pairs signed-rank test: *P < 0.05; **P < 0.01.

As CCR5 genetic mutations proved to be rare in the controller group, we explored other possible causes for CCR5 downregulation in HIV-specific CD4 + T cells. Considering that specific CD4 + T cells of controllers showed increased expression of the CCL5/RANTES chemokine in single-cell analysis (Fig. 2), we asked whether production of β-chemokines upon Gag stimulation could lead to downregulation of CCR5 in an autocrine fashion. To this goal, CD8-depleted patient PBMC (HIC *n* = 8; ART *n* = 8) were stimulated for 3 days with HIV antigens consisting either of the Gag293 peptide alone, or in a pool of immunodominant Gag peptides (Fig. 7a). Interestingly, CCR5 expression in memory CD4 + T cells decreased upon stimulation with Gag293 in the HIC group (*P* < 0.01), while this was not the case in the ART group (*P* > 0.05) (Fig. 7b and Supplementary Fig. 7a). Stimulation with a Gag peptide pool induced a more marked CCR5 downregulation than with Gag293 alone, with again a significant decrease reached in the HIC but not in the ART group (Fig. 7b). The exclusion of one outlier made the decrease significant in the ART group (*P* < 0.05), suggesting that CCR5 downregulation may occur in both patient groups upon strong Gag antigenic stimulation.

The addition of a cocktail of antibodies blocking the main CCR5 β-chemokine ligands (CCL3, CCL4, and CCL5) during Gag-specific stimulation abrogated the CCR5 decrease seen in the HIC group, supporting the notion of a chemokine-dependent down-regulation of CCR5 (Fig. 7b). Potent TCR stimulation with superantigens (SEA + SEE) caused a marked CCR5 downregulation in both groups, that was only partially restored by chemokine blocking. Of note, chemokine blocking also increased CCR5 expression in unstimulated cells (Supplementary Fig. 7b, c), pointing to a degree of tonic CCR5 downregulation by β-chemokines present in human plasma. Partial inhibition of CCR5 downregulation upon Gag stimulation could also be observed in the presence of a CCL5 blocking antibody alone, indicating that CCL5 production contributed to CCR5 downregulation (Supplementary Fig. 8a). Blocking CCL3 also led to restoration of high CCR5 surface expression while blocking CCL4 showed no effect (Supplementary Fig. 8b), suggesting that the CCL3 chemokine also contributed to CCR5 downregulation. Measurement of β-chemokines released in culture supernatants after antigenic stimulation (Supplementary Fig. 9a) or circulating in patient plasma (Supplementary Fig. 9b) did not show significant differences between groups. However, we noted a trend for increased secretion of both CCL3 and CCL5 in supernatants of Gag-stimulated cultures from the HIC group, consistent with a possible role of these two β-chemokines in CCR5 downregulation.

To determine whether low CCR5 expression upon antigenic stimulation resulted from a mere internalization or a degradation of the receptor, we stimulated patient PBMC with a Gag peptide pool and monitored CCR5 expression either by classic surface labelling post-stimulation or by antibody feeding, which consisted in incubating the cells with the fluorescently labelled anti-CCR5 antibody for the whole duration of an 18 h stimulation (Supplementary Fig. 10). Antibody feeding enabled the detection of the pool of CCR5 receptors that bound the antibody and then internalized during the stimulation period, in addition to the receptors that remained at the cell surface. Comparison of the two conditions showed that CCR5 expression decreased upon antigenic stimulation when monitored by surface labeling, but remained unchanged upon antibody feeding (Supplementary Fig. 10a), indicating that CCR5 downregulation resulted from internalization without significant degradation of the internalized receptors. CCR5 surface expression decreased in the whole CD4 + CD45RA− population in the 4 HIC patients tested (*P* = 0.05) but in only two out of 4 ART patients tested (*P* = 0.47), while CCR5 amounts detected by antibody feedings remained stable post-Gag stimulation in all the patients tested (Supplementary Fig. 10b).

In these in vitro stimulation experiments, the subset of Gag-specific cells was detected by the induction of activation-induced markers (AIM) in memory CD4 + T cells. As shown for a representative HIC patient (Supplementary Fig. 10c), specific AIM+ cells (CD69+ CD154+) showed a trend for higher CCR5 surface expression than non-specific AIM− cells (CD69- CD154-), consistent with MHC II tetramers results (Fig. 3c). Antibody feeding led to a marked increase in CCR5 detection, both in the specific and non-specific memory CD4 + T cells populations (Supplementary Fig. 10d). The increase was the most significant in specific AIM + cells of HIC patients (*P* = 0.004), though it was also observed in specific cells of ART patients (*P* = 0.01). Taken together, these experiments suggest that CCR5 surface levels are primarily regulated by dynamic receptor internalization, both in the specific and non-specific memory CD4 + T cell populations.

We then analyzed the kinetics of CCR5 downregulation and re-expression in memory CD4 + T cells upon Gag antigenic stimulation. The decrease in CCR5 expression persisted until day 6–7 post-stimulation in 2/2 controllers and 1/2 treated patients tested (Supplementary Fig. 7d). Superantigen stimulation also led to a marked CCR5 downregulation until day 6–7, prior to rebound. Interestingly, repeated Gag stimulation every 3 days limited CCR5 rebound in the two controllers, and showed a partial effect in one of the treated patients. These findings support a role for chronic antigenic stimulation in driving persistent CCR5 downregulation, particularly in the controller group.

### Strong TCR signals induce CCR5 downregulation

**Fig. 8:**
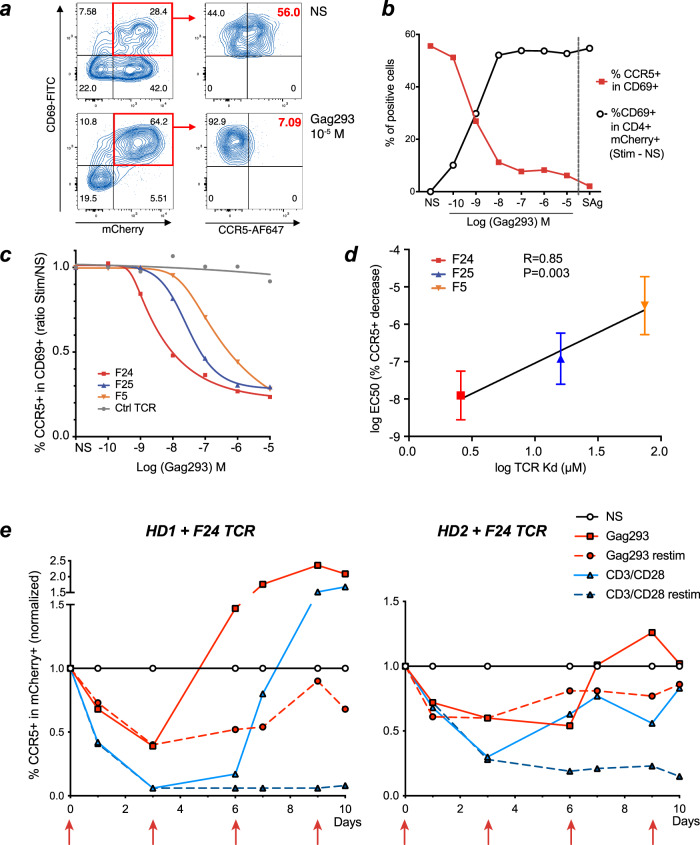
Strong TCR signals induce CCR5 downregulation. **a**–**d** Analysis of CCR5 downregulation in HD PBMC transduced with Gag293-specific TCRs and activated with Gag293-loaded APC. **a** Gating strategy: TCR-dependent activation was measured by CD69 induction in TCR-transduced (mCherry+) CD4 + T cells (left). CCR5 downregulation was measured in activated mCherry+ CD69 + CD4 + T cells (right). NS Non-stimulated. **b** Example of quantitation of T cell activation (%CD69 + in Stim - NS Black curve) and of CCR5 down-regulation (%CCR5+ in CD69+) in TCR F24-transduced PBMC, in function of Gag293 concentration. Superantigens (SAg) were used as positive control. **c** Analysis of CCR5 downregulation as in B, for PBMC transduced with 3 Gag293-specific TCRs of increasing affinity (F5 < F24 < F24) or with a control flu-specific TCR (Ctrl) in an HLA-DR15 context. One representative experiment out of *n* = 3 is shown. The % CCR5 + cells in CD69 + mCherry+ CD4 + T cells is normalized to that measured in the unstimulated condition (NS). **d** Correlation between TCR affinity (Kd) for the Gag293/HLA-DR15 complex and the Gag293 EC50 concentration for CCR5 downregulation (as determined in **c**). Means ±SD are shown for *n* = 3 experiments. The linear regression coefficient R and associated *P*-value for the slope being significantly ≠ 0 are reported. **e** Kinetics of CCR5 downregulation in TCR transduced CD4 + T cells upon antigenic stimulation. TCR-transduced CD4 + T cells (mCherry+ CD4 +) were monitored for CCR5 expression after stimulation at day 0 with either the cognate peptide (Gag293; solid red lines) or anti-CD3/anti-CD28 coated beads (CD3/CD28; solid blue lines). Kinetics are shown in transduced cells of *n* = 2 healthy donors, HD1 (left), and HD2 (right). The proportion of CCR5 + cells as compared to the unstimulated condition (NS) is reported. In the “restim” conditions (dashed lines), cultures were restimulated at days 3, 6, and 9, as indicated by red vertical arrows.

As we previously documented the presence of TCRs of particularly high affinity in HIV-specific CD4 + T cells of controllers^17^, we reasoned that stronger TCR signals in the controller group may contribute to CCR5 downregulation. To test this notion, we transduced HD PBMC with a high-affinity Gag293-specific TCR (F24) derived from a controller patient and stimulated these cells with autologous APC pulsed with increasing doses of the Gag293 peptide. As expected, we observed a dose-dependent increase in CD4 + T cells expressing the CD69 activation marker among TCR-expressing mCherry+ cells (Fig. 8a, b). Conversely, CCR5 expression showed a dose-dependent decrease among the activated CD69 + mCherry+ CD4 + T cells (Fig. 8b). Chemokine blocking antibodies partially inhibited CCR5 downregulation (Supplementary Fig. 8c, d), indicating that β-chemokine secretion contributed to the TCR-dependent modulation of CCR5, but not ruling out TCR direct effects. Comparison of CCR5 modulation induced by Gag293-specific TCRs of increasing affinity (F5 < F25 < F24) showed that high-affinity TCRs downregulated CCR5 at lower antigen dose (Fig. 8c). Indeed, TCR affinity correlated with the EC50 for CCR5 downregulation (*R* = 0.85, *P* < 0.005, Fig. 8d). Taken together, the high-affinity TCRs characteristic of HIV controller Gag-specific CD4 + T cells proved more efficient at inducing CCR5 downregulation than medium/low-affinity TCRs, providing a mechanistic explanation for the lower expression of CCR5 in controller specific CD4 + T cells. Furthermore, analysis of the kinetics of CCR5 downregulation in TCR-transduced cells showed that chronic antigenic stimulation could maintain the decrease in CCR5 expression over time (Fig. 8e).

## Discussion

This study provides evidence for low CCR5 expression in HIV-specific CD4 + T cells of HIV controllers, a property that results in decreased susceptibility to R5 HIV entry. An inverse correlation was observed between CCR5 expression in HIV-specific CD4 + T cells and their frequency in peripheral blood, supporting the notion that low CCR5 expression protects these cells from depletion in vivo. We propose that this straightforward mechanism directly contributes to the establishment of HIV control. Protection of the limited but key population of HIV-specific CD4 + T cells can account for the preservation of the total CD4 + T cell population, through help provided to the different arms of the antiviral immune response, which in turn contain HIV replication and prevents widespread CD4 + T cell depletion. Gene therapy experiments in the SIV/macaque model have indeed demonstrated that protection of a limited subset of CD4 + T cells from infection is sufficient to maintain a broader CD4 + T cell population through bystander effect^32^. HIV-specific CD4 + T cells are thought to be preferentially infected by HIV as compared to CD4 + T cells of other specificities^33^, and are rapidly depleted in the course of progressive HIV infection, to reach almost undetectable frequencies in persistently viremic patients^34^. In addition, HIV-specific CD4 + T cells are only partially recovered in treated patients and retain suboptimal function, pointing to a failure in immune reconstitution^9^. HIV controllers represent the exception, with a preservation of the specific CD4 + T cell population not only in frequency but also in terms of quality. Protection of this highly functional CD4 + T cell population may be sufficient to sustain a fully competent antiviral adaptive response and thus promote HIV control.

The mechanism involved in limiting CCR5 expression had a clear genetic origin in two cases. Patient HIC11 harbored bi-allelic CCR5 mutations that severely compromised, but did not abrogate, CCR5 surface expression. The combination of the CCR5 Δ32 and Q280P mutations resulted in very low but still detectable capacity to sustain R5 HIV fusion, a phenotype that is consistent with a capacity to limit HIV spread, but not to prevent HIV acquisition. We then identified a second controller patient with bi-allelic mutations that markedly impaired CCR5 surface expression, indicating that patient HIC11 was not a unique case. It is interesting to note that cases of bi-allelic CCR5 mutations, such as homozygous Δ32 deletions, or the combination of a Δ32 deletion with a C101X nonsense mutation, have also been reported in highly exposed non-infected individuals^27,35^. Our findings highlight, for the first time to our knowledge, that bi-allelic CCR5 mutations can not only prevent HIV acquisition, but also promote natural HIV control. The possibility remains that other CCR5 genetic polymorphisms limited HIV replication in the group of controllers studied. A meta-analysis of genetic studies showed a genome-wide significant association between the CCR5 locus and HIV viral load^36^. In particular, CCR5 promoter haplotypes resulting in low transcriptional activity were associated with a decreased rate of HIV disease progression in early studies^31,37^. However, we did not observe an accumulation of protective CCR5 promoter haplotypes, such as HHA or HHC, in the admittedly limited group of controllers studied. Differences in CCR5 mRNA levels did not reach significance between the HIC and ART groups, though a trend for lower CCR5 mRNA expression was noted in Tet+ cells of controllers compared to those of treated patients. Interestingly, a recent study suggested that a 500 kb region encompassing the CCR5 locus had lower levels of active transcription in a subset of HIV controllers, through mechanisms that may have a genetic basis^38^. It is thus possible that negative transcriptional regulation contributes in part to low CCR5 expression in HIV-specific CD4 + T cells of controllers. However, differences were clearer at the CCR5 protein expression level, pointing to additional post-transcriptional mechanisms involved in limiting CCR5 expression.

Several elements suggest a role for increased β-chemokine secretion in driving CCR5 downregulation in controller specific CD4 + T cells: (1) the transcriptional analysis revealed a significantly increased expression of the chemokine CCL5 in controller Tet+ cells; (2) Gag-specific stimulation of patient CD4 + T cells in vitro led to a more marked CCR5 downregulation in the HIC than the ART group; (3) repeated stimulation with Gag antigens induced persistent CCR5 downregulation; (4) the antigen-induced CCR5 downregulation could be reverted by a cocktail of anti-β-chemokines antibodies, demonstrating that autocrine or paracrine chemokine secretion was involved in CCR5 downregulation. In addition, we could show in TCR transfer experiments that TCRs of higher affinity induced more potent CCR5 downregulation, thus linking the strength of TCR-dependent signals to CCR5 modulation. Therefore, we propose that the high TCR affinity characteristic of controller Gag-specific CD4 + T cells results in a more abundant β-chemokine production upon viral antigen stimulation, in turn leading to CCR5 internalization and protection from HIV entry.

Chemokine blocking experiments suggested that both CCL3 and CCL5 contributed to CCR5 downregulation. It should be noted that the antibodies and primers used in the present study did not distinguish between CCL3 and its variant CCL3L1. Therefore, CCL3L1 may also play a role in CCR5 downregulation, considering, in particular, its higher affinity for CCR5 compared to that of CCL3. It may be relevant that CCL3L1 shows marked gene copy number variations in the human population, and that low CCL3L1 copy numbers have been previously associated with faster progression to AIDS in some though not all studies^39–41^. We also obtained evidence for an involvement of the CCL5 chemokine in CCR5 downregulation, based on blocking experiments but also on an increased expression of CCL5 transcripts in controller-specific CD4 + T cells. The chemokine CCL5, to the difference of other CCR5 ligands, is stored in intracellular granules and released rapidly upon TCR stimulation^42^, which may facilitate the protection of CCL5hi HIV-specific CD4 + T cells that form immunological synapses with infected cells. It is also relevant that CMV-specific CD4 + T cells, which show advanced Th1 differentiation, were proposed to escape HIV infection due to high autocrine β-chemokine production^43^. A similar mechanism appears to be at work in HIV-specific CD4 + T cells of controllers, which are endowed with high β-chemokine production capacity and show signs of advanced Th1 differentiation, including a correlated expression of CCL5 with cytotoxic mediators (this study), and better IFN-γ secretion and degranulation capacity^9^. Studies in mouse models have linked high TCR affinity to preferential Th1 differentiation^44,45^, supporting a key role of TCR determinants in defining β-chemokine production capacity and ultimately controlling CCR5 surface expression.

Strong signals via the TCR/CD3 complex can downregulate CCR5 through chemokine-dependent receptor internalization, as shown by CCR5 antibody feeding experiments, but also in part through inhibition of CCR5 transcription^46,47^, which may account for the trend for lower CCR5 mRNA expression in controller Tet+ cells. Conversely, cytokines produced at a late stage of T cell activation, such as IL-2 and IL-4, can increase CCR5 transcription^27^. Importantly, we show that repeated antigenic stimulation can prevent the rebound in CCR5 expression associated with late-stage T cell activation, suggesting that persistence of a limiting amount of viral antigens may be sufficient to maintain CCR5 downregulation in the long term. Inflammatory cytokines such as IL-15 also lead to CCR5 transcriptional upregulation in T cells^48^. Thus, a lower level of chronic immune activation in the HIC than in the ART group, as suggested by lower PD-1 and higher IL-7R expression, may also contribute to the generally decreased CCR5 expression levels in memory CD4 + T cells of controllers. Strong TCR signals and low inflammation are not mutually exclusive, and may rather synergize in limiting CCR5 expression. Indeed, strong TCR signals promote the helper and cytotoxic functions of HIV-specific CD4 + T cells^17,18^, in addition to driving CCR5 downregulation; this may result in a better containment of viral replication, and thus in a decrease of inflammatory cytokines associated with progressive HIV infection, further limiting CCR5 expression.

High TCR affinity characterizes the HIV-specific but not the non-specific CD4 + T cell population in controllers^16^, which may help explain why CCR5 expression is proportionately more decreased in specific than in non-specific cells of controllers compared to those of treated patients. Autocrine CCR5 downregulation in β-chemokine producer cells is likely more efficient than paracrine CCR5 downregulation in neighboring cells, though the in vitro stimulation assay suggests that paracrine effects may also occur. It is also important to note that not all controllers showed signs of CCR5 downregulation. This may account for the divergent findings in the literature, with some^38,49^ but not all studies^22,50^ reporting cases of low CCR5 expression in controller CD4 + T cells. In the present study, a few patients achieved efficient viral control in spite of high CCR5 expression in memory CD4 + T cells, implying that CCR5 downregulation is not the unique mechanism underlying natural HIV control. It was noteworthy that controllers with high CCR5 expression had undetectable Gag293-specific CD4 + T cells, and more generally that the frequency of Gag293-specific cells inversely correlated with CCR5 expression, suggesting that CCR5 downmodulation is a key parameter involved in the persistence of the antiviral CD4 + T cell response.

The fact that CCR5 downregulation was significant in the CM but not in the EM Gag293-specific population of controllers is relevant, considering the essential role assigned to the CM subset in HIV/SIV pathogenesis^51^. In the model of natural SIV infection of sooty mangabeys, the CM CD4 + T cell subset is thought to escape depletion due to a particularly low CCR5 expression, resulting in preserved CD4 + T cell renewal capacity and lack of disease progression^52^. A similar mechanism has also been proposed to account for the preservation of CM CD4 + T cells in viremic non-progressing HIV-infected children^53^. Our findings in natural HIV controllers support the idea that sparing CM CD4 + T cells through CCR5 downregulation is a central theme in non-pathogenic HIV/SIV infection.

In conclusion, HIV-specific CD4 + T cells of controllers proved less susceptible to HIV entry due to negative regulation of the CCR5 coreceptor. TCR-dependent mechanisms and rare genetic mutations converged in decreasing CCR5 expression, suggesting a role for CCR5 downregulation in natural HIV control. Recently, adoptive cellular therapy of CCR5 knock-out autologous T cells was shown to induce long-term HIV control in a subset of treated patients^54,55^. Thus, mimicking natural HIV control through CCR5 inactivation or downregulation represent promising approaches towards a functional HIV cure.

## Methods

### Study design

HIV controllers (HIC group; *n* = 25) were recruited through the CO21 CODEX cohort set up by the Agence Nationale de Recherche sur le SIDA et les Hépatites Virales (ANRS). HIV controllers were defined as HIV-1-infected patients who had been seropositive for >5 years, who had received no antiretroviral treatment, and for whom >90% of plasma viral load measurements were undetectable by standard assays. All HIV controllers included in the study had viral loads of <50 copies/ml at inclusion. Ultrasensitive viral load measurements were performed as previously described^56^. The group of efficiently treated patients (ART group; *n* = 24) had received antiretroviral therapy for a minimum of 5 years and showed long-term HIV-1 suppression with viral loads of <50 copies/ml. Treated patients were recruited at the Raymond Poincaré and Bicêtre hospitals (France). Patients were included in the MHC II tetramer study (HIC *n* = 12; ART *n* = 15) if their genotype matched at least one of the following alleles: DRB1*0101, DRB1*0401, DRB1*0405, DRB1*0701, DRB1*1101, DRB1*1302, or DRB1*1502 (Supplementary Table 1). Healthy donors were anonymous volunteers who donated blood at the Etablissement Français du Sang. The study was promoted by ANRS and approved by the Comité de Protection des Personnes IDF-VII under number 11–33. All participants gave written informed consent prior to inclusion in the study.

### Cell culture

Peripheral blood mononuclear cells (PBMC) were isolated from heparinized blood via density gradient centrifugation on Ficoll-Paque PLUS (GE Healthcare Life Sciences) and were either cryopreserved or used freshly for the preparation of monocyte-derived dendritic cells (MDDC). PBMC were cultivated in RPMI-1640 medium supplemented with 10% heat-inactivated fetal calf serum (FCS), 100 U/ml penicilline and 100 ug/ml streptomycin (complete RPMI). MDDC were obtained by positive selection of CD14 + monocytes using magnetic microbeads (Human monocyte isolation kit II, Miltenyi Biotec). Monocytes were plated at 2 × 10^6^ cells/mL in synthetic AIM-V medium (Life Technologies) supplemented with 10 ng/mL GM-CSF and 20 ng/mL IL-4 (both from Miltenyi Biotec) and incubated for 5–7 days at 37 °C in a 5% CO_2_ incubator. Differentiated immature MDDC were collected and cryopreserved until further use in antigen presentation experiments.

### MHC II tetramer labeling and single-cell sort of HIV-specific CD4 + T cells

#### MHC II tetramer loading

Patients were genotyped for the HLA-DRB1 gene at a 4-digit resolution using the INNO-LiPA HLA-DRB1 Plus kit (Fujirebio). APC-labelled MHC-II tetramers loaded with Gag293 peptide (FRDYVDRFYKTLRAEQASQE) were obtained through the NIH Tetramer Core Facility at Emory University (Atlanta, GA) for the DRB1*0101, DRB1*0401, DRB1*0405, DRB1*0701, DRB1*1502, and DRB5*0101 alleles. The DRB1*1101 and DRB1*1302 biotinylated monomers were obtained through the Tetramer Core Laboratory of the Benaroya Research Institute (Seattle, WA). Monomers were loaded with 0.2 mg/ml peptide by incubation at 37 °C for 72 h in the presence of 2.5 mg/ml *n*-octyl-β-d-glucopyranoside and protease inhibitors. Peptide-loaded monomers were tetramerized using APC-conjugated streptavidin (eBioscience). For each tetramer loaded with the Gag293 peptide, a corresponding control tetramer was loaded with an irrelevant peptide (the CLIP peptide PVSKMRMATPLLMQA).

#### Single cell sorting

For the detection of Gag293-specific CD4^+^ T cells, patient PBMC were labelled with MHC-II tetramers as follows: at least 10^7^ PBMC per sample were incubated with 1 µg MHC-II tetramer/10^6^ cells at a concentration of ≥1 µg/ml in RPMI medium supplemented with 15% human AB serum for 90 min at 4 °C. Antibodies for surface markers were added for the last 30 min of labelling, using the antibody combination detailed in Supplementary Table 3. Gag293-specific CD4 + T cells (Tet+) were gated on the following populations: Viable, CD14−, CD20−, CD3+, CD8−, CD4+, and tetramer+. Non-specific memory CD4 + T cells (Tet−) were gated on the Viable, CD14−, CD20−, CD3+, CD8−, CD4+ , tetramer−, CD45RA− population. Tet+ and Tet− populations were single-cell sorted using a FACSAria II cell sorter (BD Biosciences) placed under a microbiological safety cabinet. Each cell was sorted into a single well of a 96-well PCR plate containing 0.5% NP40 lysis buffer, an RNase inhibitor (SUPERase-in, Thermo Fisher Scientific), and 5x reverse-transcription buffer (Superscript VILO cDNA synthesis kit, Thermo Fisher Scientific). For each of 9 HIC and 9 ART patients with detectable Tet+ cells, 25 Tet+ and 25 Tet− cells were sorted, using the single-cell purity mode to avoid doublets. The “index sorting” mode was used to record the individual fluorescence parameters of each sorted cell. In addition to the parameters used for cell gating, we recorded 4 additional parameters for in-depth phenotyping of the Tet+ and Tet− populations (CCR7, CCR5, CXCR3, and PD-1). The sorted and lyzed cells were stored at −80 °C until use. At least 5 × 10^6^ events were also recorded during sorting to phenotype the different CD4 + T cell populations.

### Phenotyping of the total CD4 + T cell population

Frozen PBMC were thawed and washed twice with PBA buffer (phosphate buffer saline, 1% bovine serum albumin, 10 mM NaN3). Cells were then stained with the antibody combination reported in Supplementary Table 4, in a final volume of 100 uL PBA for 30 min at 4 °C. Cells were washed 2x in PBA, fixed with 2% paraformaldehyde (PFA), and acquired on a FACSymphony flow cytometer (BD Biosciences) with the FACSDiva software v8.0 (BD Biosciences). Analysis was carried out with the FlowJo v10.4 software.

### Single cell multiplexed qPCR

#### Reverse transcription and pre-amplification of genes of interest

Lyzed Tet+ and Tet− single-cell samples were thawed, denatured for 90 s at 60 °C, and reverse transcribed with the SuperScript III reverse transcriptase. A total of 48 genes of interest were analyzed in every single cell using the Biomark microfluidics system (Fluidigm). The 48 PCR primer pairs used, reported in Supplementary Table 2, were first tested by classical RT-qPCR and optimized to minimize non-specific amplification and primer dimers. The single-cell lysates were then processed for a first PCR in the presence of a mix of the 48 primer pairs at a concentration of 50 nM in presence of the TaqMan PreAmp Master Mix containing the AmpliTaq Gold DNA Polymerase (Thermo Fisher Scientific). After enzyme activation (95 °C 10 min), twenty amplifications cycles (96 °C 5 s, 60 °C 4 min) were performed followed by exonuclease I treatment to remove unincorporated primers. Amplified cDNA were then diluted 1:5 in TE buffer and stored at 20 °C.

#### Multiplexed qPCR

Samples and primers were loaded in a 48.48 Fluidigm Dynamic array (Fluidigm) according to the supplier recommendations. Briefly, each primer pair was diluted at 10 μM and mixed with the 2x assay loading reagent. Amplified cDNA samples were mixed with Evagreen dye and the 20x sample reagent. Samples and primers were then loaded in the primed 48.48 Fluidigm Dynamic array, and real-time PCR was run on the Biomark system. The data was collected and processed using the “Fluidigm real-time PCR Analysis” software v2.1. Cells showing low level of expressions of the GAPDH housekeeping gene (GAPDH Ct<18) were removed. Genes showing weak expression due to primer dimer accumulation, as visualized on the melting curve, were also removed. Expression of each gene was quantified as number of specific transcripts reported to those of GAPDH x 10^3^. Analysis of index-sorted flow cytometry parameters was run in parallel after compensation based on mono-stained cells. The resulting dataset consisted of 6 mean fluorescence intensity (MFI) parameters measuring protein expression and 47 relative gene expression parameters measuring mRNA abundance for each of 740 analyzed single cells.

### Functional analysis of HIV fusion

#### Production of BlaM-Vpr HIV-1 particles

Beta-lactamase-Vpr (BlaM-Vpr)-containing HIV-1 particles were generated to assay HIV-1 fusion, as described by Cavrois et al.^28^. The two HIV-1 proviral clones used, which expressed either the X4 Env from HIV-1 NL4-3 or the R5 Env from HIV-1 JR-FL, were a kind gift from Dr. Bernard Lagane (CPTP, Toulouse, France), and have been reported previously^57^. These proviral clones, which are derived from pNL4-3, carry a luciferase reporter gene in place of the nef gene, and differ in a fragment encompassing the env gene between positions 6113 and 8797. To produce BlaM-Vpr HIV-1 particles, 8 × 10^6^ HEK 293 T cells in 162 cm^2^ culture flasks were cotransfected using the calcium phosphate-DNA co-precipitation method with 60 μg proviral DNA, 20 μg pCMV-BlaM-Vpr plasmid, and 10 μg pAdVAntage vector (Promega), which increases transient protein expression. The culture media was replaced 18 h post-transfection, and supernatants containing viral particles were harvested at 48 h. The supernatants were clarified at low speed (1460 g) and then ultracentrifuged at 23,000 g for 90 min at 4 °C. The pelleted viruses were resuspended in DMEM 10% FBS at a final 50x concentration, quantified for their content in HIV-1 Gag p24 antigen (Alliance HIV P24 antigen ELISA Kit from PerkinElmer), and stored at −80 °C before use.

#### Fusion assay

HIV-1 fusion was monitored by the cleavage of the fluorogenic substrate coumarin cephalosporin fluorescein (CCF2) by the beta-lactamase (BlaM) released from incoming BlaM-Vpr virions. Cleavage of CCF2 results in a loss of fluorescence resonance energy transfer (FRET) between the coumarin and the fluorescein moieties of the CCF2 molecule^28^. Target cells consisting of unstimulated PBMC and BlaM-Vpr virions were mixed in the following proportions: 10^6^ cells in 50 uL with 150 ng p24 of HIV-1 JR-FL-BlaM-Vpr or 30 ng p24 of HIV-1 NL4-3-BlaM-Vpr in 50 uL. The number of target cells used per assay was 10^6^ PBMC for an analysis of fusion in total CD4 + T cells but was increased to 10^7^ - 4 × 10^7^ PBMC for analyses in Tet+ CD4 + T cells. For analyses of CCR5 mutants, target cells consisted of 0.5 × 10^6^ CCR5-nucleofected PBMC. The BlaM-Vpr virus/cell mix was spinoculated (2000 g, 1 h, 4 °C), and then incubated for 3 h at 37 °C. At the end of the incubation period, cells were centrifuged and resuspended in 0.925 uM CCF2-AM substrate according to the manufacturer’s instructions (Life Technologies). Cells were kept in the dark at room temperature for 2 h and washed twice with PBS. Cells were then either labelled with MHC II tetramers followed by the antibody combination shown in Supplementary Table 5 or directly labeled with the antibody combination shown in Supplementary Table 6, following procedures described above. Cells were then fixed in PFA 2% and acquired on a FACSymphony flow cytometer (BD Biosciences). Fusion was monitored by a shift in fluorescence of the CCF2-AM substrate, which upon excitation at 405 nm emits at 520 nm in its intact form and at 447 nm in its cleaved form.

### Sequencing of the CCR5 locus

#### Detection of the CCR5 *Δ*32 mutation

Genomic DNA was extracted from patient PBMC using a Qiagen kit and tested for the presence of the Δ32 deletion by PCR. The PCR reaction was performed using Taq Platinum (Thermo Fisher Scientific) in presence of the following primers: Forward 5′-CTT CAT TAC ACC TGC AGC TCT-3′; Reverse 5′-CAC AGC CCT GTG CCT CTT CTT C-3′. The PCR product was run on 2% agarose gel stained with ethidium bromide and analyzed for its size, with a 183 bp fragment expected for WT CCR5 and a 151 bp fragment for CCR5 Δ32.

#### Long-read sequencing

The CCR5 locus, ranging from −5472 in the promoter region to 3734 in the 3′ UTR, according to the numbering system of Mummidi et al. where + 1 is the first nucleotide of the translational start site^58^, was sequenced using the PacBio long-read technology (Pacific Biosciences). The CCR5 locus was first amplified from genomic DNA in two overlapping 5.7 kb PCR fragments using the Q5 Hot Start High-Fidelity DNA polymerase (New England Biolabs) with the following primers: Forward#1 5′-GCA TGG GAA AAG TCA GGAT TGA AA-3′; Reverse#1 5′-ACA CCA GTG AGT AGA GCG GA-3′; Forward#2 5′-GAG CTG AGA CAT CCG TTC CC-3′; Reverse#2 5′-ATG TGC CTA CAA CTC AGG GC-3′. PCR products were checked on a BioAnalyzer (Agilent), purified with AMPure PB beads (Pacific Biosciences), and marked with different 16-base barcodes. The pooled products were used to generate libraries that were sequenced using single-molecule real-time (SMRT) sequencing according to the manufacturer’s instructions. Briefly, the template libraries were purified, treated for repair of DNA ends, and converted to circular templates by the ligation of hairpin “bell” adapters using the SMRTbell Barcoded Adapter complete Prep kit (Pacific Biosciences). The sequencing technology relied on incorporation of fluorescent nucleotides onto the circular templates by immobilized single DNA polymerase molecules. As the data was collected in real-time during rolling-circle replication, each template was read multiple times, resulting in a consensus sequence that limited systematic error. Sequencing was performed on a Sequel instrument using the SMRT Link v5.1 software. The multiplexed libraries were analyzed in an SMRT Cell 1 M v2, using the Sequel Sequencing kit 2.1 with a 600 min movie time. Data was processed with the SMRT Link v5.1 package: the SMRT raw reads were demultiplexed using the Demultiplex Barcodes application; the quality-filtered reads were then selected in the SMRT Analysis application to include only circular consensus sequences (CCS) > 100 bases with at least 5 complete passes and 0.99 minimum predicted accuracy. The final average number of CCS reads distributed on the two overlapping 5.7 kb fragment was about 1500. Those CCS reads were used for further analyses including variant detection, as explained in the Statistical and bioinformatics analyses section.

#### V3 Env sequence analysis

The V3 region of HIV-1 Env was sequenced as previously described^59^ and tropism was determined by Geno2Pheno algorithm v2.5, with a False positive rate of 10% (https://coreceptor.geno2pheno.org).

### Analysis of CCR5 mutants

#### Construction of mutant CCR5 clones

To characterize the CCR5 mutants at a functional level, CCR5 ORFs were cloned into the lentiviral expression vectors pCDH-EF1α-MCS-T2A-GFP or pCDH-EF1α-MCS-T2A-mCherry (System Biosciences). The CCR5 ORFs were amplified from genomic DNA with primers which added a BamHI and a NotI restriction sites in 5′ and 3′, respectively: Forward primer 5′-GCC CGG GGA TCC TGG AAC AAG ATG GAT TAT CAA GTG-3′; Reverse primer 5′-TAA TGC GGC CGC CAA GCC CAC AGA TAT TTC CT-3′. PCR products were purified and first cloned into a Zero Blunt TOPO vector (Thermo Fisher Scientific). Clones carrying the mutants of interest were double-digested by BamHI and NotI and resulting fragments were cloned into the multiple cloning site (MCS) of pCDH lentivectors. The sequence of the resulting mutant CCR5 inserts was confirmed by Sanger sequencing (Eurofins Genomics).

#### Nucleofection of CCR5 mutants

PBMC were thawed and rested for 2 h at 37 °C in complete RPMI without antibiotics, washed with PBS, and resuspended in Buffer T (Neon Transfection 100 µL kit, Invitrogen, Carlsbad, CA) at a concentration of 22.2 × 10^6^/mL. For each condition, 5 × 10^6^ cells (225 uL) were transferred in an eppendorf tube and mixed with 25 uL of purified CCR5 plasmid at 1 mg/ml in water. PBMC were nucleofected using the Invitrogen Neon apparatus, using 100 uL Neon tips under optimized conditions (2400 V; 10 ms pulse; 2 pulses). A total of 4 × 10^6^ nucleofected PBMC (corresponding to two nucleoporations of 100 uL) were resuspended in 2 mL complete RPMI without antibiotics, and incubated for 24 h at 37 °C under 5% CO_2_. Nucleofected cells were tested at day 1 in HIV-1 fusion assays, as described above. The expression of CCR5 WT and mutant plasmids was analyzed by flow cytometry in the naive CD4 + T cell population, which expresses only minimal levels of endogenous CCR5. Specifically, CCR5 expression and fusion frequency were measured in cells labelled with the antibody combination shown in Supplementary Table 7, with transfected naive CD4 + T cells gated as viable CD3 + CD4 + CD45RA + CCR7 + mCherry+ cells. Events were acquired on a FACSymphony flow cytometer (BD Biosciences).

#### Intracellular labeling of tagged CCR5 mutants

WT and mutant CCR5 coding sequences were tagged with a FLAG sequence in N-terminal position as described in ref. ^60^. HEK 293 cells were transfected with WT and mutant CCR5 using Lipofectamine 2000 (Life Technologies). Transfected cells were detached at day 2 and fixed in 4% paraformaldehyde for 10 min at room temperature (RT). The total amount of CCR5 in the cells was determined after permeabilization of fixed cells with 0.05% saponin in PBS with BSA 3% for 10 min at RT. Unpermeabilized cells were used to measure CCR5 surface expression. Cells were incubated with an antibody against the FLAG epitope (anti-Flag M1 used at 1:500, Sigma-Aldrich) followed by a PE-conjugated goat anti-mouse secondary antibody (used at 1:100, BD Biosciences). The stained cells were washed and analyzed on a Cytoflex S flow cytometer (Beckman Coulter) using the CytExpert Sofware v2.4.

#### Generation of CCR5 knock-out primary CD4 + T cells

The CRISPR-Cas9 ribonucleoprotein (RNP) knock-out protocol was adapted with some modifications from two previously published articles^61,62^. Briefly, human PBMCs were isolated by ficoll gradient centrifugation and CD4 + T cells were sorted by positive selection using magnetic beads (Human CD4 + T cell Isolation kit; Miltenyi Biotec). CD4 + T cells were cultured at 1 × 10^6^ cells/mL in complete RPMI medium complemented 100 U/mL IL-2 (R&D Systems). T cells were activated with 10 uL/mL of T Cell TransAct (Miltenyi Biotec) for 72 h prior to nucleofection. The CCR5 gene was targeted by a combination of 3 crRNA to optimize gene knock-out frequency: CCT GAC AAT CGA TAG GTA CC; AAC ACC AGT GAG TAGAGC GG; ACA ATGTGT CAA CTC TTG AC. Chemically modified crRNA (Alt-R crRNA) with increased nuclease resistance were obtained from Integrated DNA technologies (IDT). Each CCR5 targeting Alt-R crRNA, negative control crRNA, and Alt-R CRISPR-Cas9 tracrRNA (all from IDT) was resuspended at 200 uM in IDT Duplex Buffer. For duplexes formation, oligos were mixed at an equimolar concentration in sterile PCR tubes and annealed for 5 min at 95 °C in a thermocycler and slowly cooled down to room temperature. RNP complexes were formed by mixing, per-transfection, 0.25 uL of each of the three crRNA-tracrRNA duplexes (equal to 25 pmol each, a total of 75 pmol) with 1 uL (30 pmol) of TrueCut Cas9 Protein v2 (Thermo Fisher Scientific) and incubating the mix at RT for at least 10 min. For nucleofection, 2 × 10^6^ CD4 + T cells were resuspended in 20 uL of P3 Primary Cell Nucleofector Solution (Lonza) and mixed with 1.75 uL of RNP mix and 0.5 uL (90 pmol) of 180 uM Alt-R Cas9 Electroporation Enhancer (IDT). Cells were nucleofected in a 4D-Nucleofector System (Lonza) using the P3 Primary Cell 4D-Nucleofector X Kit S and program FI-115. After nucleofection, CD4 + T cells were transferred to a 24 well plate in 2 mL of complete RPMI medium containing IL-2 and 10 uL/mL of T Cell TransAct, and were used after at least 10 days for CCR5 mutant nucleoporation and HIV-1 fusion assays as described above.

#### Analysis of CCR5 downregulation in stimulated patient CD4 + T cells

Frozen PBMCs were thawed and depleted of CD8 + T cells using magnetic beads (BD IMag, BD Biosciences). CD8-depleted cells were washed first with PBS, then with RPMI supplemented with 10% human AB serum, before being distributed in round-bottom 96-well culture plates at the concentration of 2.5 × 10^6^/mL. Cells were stimulated with the Gag293 peptide or with a pool of 37 Gag peptides corresponding to potential T cell epitopes (PTE) designed to cross-react with a broad array of HIV-1 strains (NIH AIDS Reagent Program), at concentration of 2.5 ug/mL. Cells treated with superantigens (SEA + SEE, 10 ug/mL) were used as positive control. Cells were treated or not with a combination of β-chemokine blocking antibodies (polyclonal antibodies from goat, R&D Systems) including anti-CCL5 (1:200 dilution, #AF-278-NA), anti-CCL4 (1:67 dilution, #AF-271-NA), and anti-CCL3 (1:20 dilution, #AF-270-NA), using a concentration at least twice higher than their neutralization dose (ND50), as per manufacturer’s instructions. Normal goat IgG (Biotechne, #AB-108-C) was used at 1:67 as negative control in conditions without blocking antibodies. Cells were cultivated for three days at 37 °C with 5% CO_2_ before analysis by flow cytometry. Cells were stained with the antibody panel shown in Supplementary Table 8 for 20 min at 4 °C, washed, fixed in 2% paraformaldehyde, and acquired on a BD LSR Fortessa flow cytometer.

For the measurement of β-chemokines released in stimulated cultures or present in patient plasma, supernatants were analyzed by ELISA assays with the following reagents: RANTES human instant ELISA kit (#BMS287-2INST, ThermoFisher Scientific), human CCL3 Quantikine ELISA (#DMA00, R&D Systems), and human CCL4 Quantikine ELISA (#DMB00, R&D Systems).

#### Analysis of CCR5 expression upon antibody feeding

CD8-depleted PBMC were plated at 10^6^/mL in 96-well round-bottom plates and were pretreated with and anti-CD40 antibody (Miltenyi Biotech, clone HB14) at 2 µg/mL for 20 min at 37 °C. Cells were then stimulated with a Gag peptide pool (2 µg/mL per peptide) or the superantigens SEA and SEE (1 µg/mL). Stimulations were done in the presence or absence of a CCR5-AF647 antibody (Biolegend, clone HEK1/85a, 1:50), to generate the “feeding” and “surface” conditions, respectively. Cells were collected 16 h later, stained with the antibody panel shown in Supplementary Table 9 for 20 min at 4 °C, washed, fixed in 2% paraformaldehyde, and acquired on an Attune NxT flow cytometer (Life Technologies) using the Attune NxT software v3.1. The expression of CCR5-AF647 was then compared in the “feeding” and “surface” conditions, within the AIM + (CD69+ CD154+) and AIM− (CD69− CD154−) memory CD4 + T cell populations.

### Analysis of TCR-dependent regulation of CCR5 expression

#### TCR transduction

TCR transfer in primary T cells was performed as previously described (Benati et al., 2016), with a few modifications. Three Gag293-specific TCRs (F24, F25, F5) and one control TCR (HA1.7) specific for an influenza virus epitope^63^ were used. The TCRs were modified by the introduction of an additional cysteine in each chain at positions T48C in TRA and S57C in TRB, which generated an additional disulfide bridge that promoted the pairing of the two transferred TCR chains, as originally described by Jakobsen and coll^64^. The modified TCR chains were cloned into a pCDH-EF1α-MCS-T2A-mCherry lentivector (System Biosciences), with the final construct containing a TRA-T2A-TRB-P2A-mCherry insert that ensured the equimolar expression of the TRA, TRB, and mCherry proteins. The lentivector stocks were titrated on HEK 293Tn cells, by transducing serial dilutions of the stocks, and counting the frequency of mCherry-expressing cells by flow cytometry. For TCR transfer into T cells transduction, healthy donor PBMC were pre-activated at 3 × 10^6^ cells/mL in complete RPMI supplemented with 5 μg/mL PHA (Sigma) and 50 UI/mL IL-2 for 48 h under 5% CO_2_. PHA blasts were collected, washed, and plated at 5 × 10^5^ cells/well in a 24-well plate in presence of 50 UI/mL IL-2, Lentiblast (OZ Biosciences), and 10^6^ transducing units of TCR lentivector stock (MOI = 2), in a final volume of 0.5 mL. Plates were centrifuged at 1,000 g for 1 h at 32 °C, then incubated overnight at 37 °C. The following day, fresh medium with 50 UI/mL IL-2 were added up to a volume of 1 mL. Three days later, transduction efficiency was evaluated by measuring the frequency of mCherry+ CD4 + T cells by flow cytometry.

#### Antigen presentation assay

TCR-transduced PBMC were grown for at least 7 days in complete RPMI with IL-2 before performing antigen presentation assays. Antigen presenting cells were either autologous MDDC or autologous adherent PBMC. APC were pulsed during 1 h at 37 °C with serial dilutions of Gag293 peptide ranging from 10^−4^ to 10^−9^ M and co-cultured at a 1:1 ratio with 5 × 10^4^ transduced PBMC in complete RPMI with IL-2. For chemokine neutralization experiments, blocking antibodies against CCL3, CCL4, and CCL5 (all from R&D Systems) were added to the culture at 10, 3, and 1 µg/mL, respectively, before APC pulsing. Positive controls were obtained by pulsing APC with 1 μg/ml Staphylococcal Enterotoxin A and E (Toxin Technology, Sarasota, FL), or by stimulating PBMC with 50 ng/mL phorbol 12-myristate 13-acetate (PMA) and 0.25 μg/ml ionomycin in the absence of APC. After overnight coculture, cells were stained with the antibody panel shown in Supplementary Table 10 and analyzed on an Attune NxT flow cytometer (Thermo Fisher Scientific). CD4 + T cells, defined as viable CD3 + CD4 + CD8− cells, were evaluated for immune activation (CD69+) and CCR5 expression.

### Statistical and bioinformatic analyses

#### Statistical analysis of single-cell gene and protein expression data

Statistical analyses were performed using the MAST (Model-based Analysis of Single cell Transcriptomics v1.0.5) package and linear modeling in the R (v3.3.3) software environment. The MAST approach is based on the hurdle model, a two-part generalized linear model adapted to bimodal and/or zero-inflated single cell gene expression data^26^. After a quality control step, 40 cells out of 740 were considered as outliers and were removed from the analyses. The expression of 47 genes was normalized relative to expression of the housekeeping gene GAPDH. The expression of 6 surface proteins was measured by the mean fluorescence intensity (MFI) collected by flow cytometry. The hurdle model was then applied to detect differences in gene expression between the HIV-specific (Tet+) and non-specific (Tet−) CD4 + T cells for each group of patients. Similarly, a linear model was applied to detect differences in protein expression. We defined a model including the patient number, the tetramer status (Tet−, Tet+), the patient group (HIC, ART), the interactions between the two last variables, and the interaction between the patient number and the patient group. The last interaction was useful to account for the matching of cells coming from the same patient. To test the difference in expression for each gene, we defined contrast vectors and then applied Wald tests. The resulting *p*-values were adjusted using the Benjamini and Hochberg procedure^65^. All statistical tests were two-sided.

#### Gene co-expression networks

Co-expression networks were inferred using the “huge” R package (v1.2.7)^66^ based on spearman correlations, and the clustering of the nodes was performed using mixtures of Erdos-Renyi random graphs^67^ proposed in the mixer package (v1.8). This approach allows the detection of genes communities that are positively or negatively correlated.

#### Linear discriminant analysis

A Linear Discriminant Analysis (LDA) was performed on both MFI and genes using the MASS (v7.3-47) R package (Venables and Ripley, 2002). LDA is a dimensionality reduction technique that finds a linear combination of variables (genes and MFI) that characterizes and separates the 4 subgroups of interest described by patient group (HIC, ART) and tetramer status (Tet+, Tet−). In the same manner as Principal Component Analysis (PCA), LDA looks for linear combinations of features that best explain the information contained in the data, except that LDA explicitly aims at modeling the differences between the 4 classes of interest by maximizing the variability between groups and minimizing the variability within groups. Statistical tests were done on Pearson correlations without correction for multiple comparisons. All statistical tests were two-sided.

#### Statistical analyses on bulk CD4 + T cell populations

Statistics were computed with the GraphPad Prism v7.0 software. Correlations were analyzed with Spearman’s R. coefficient. EC50 values were obtained after non-linear curve fit using a sigmoidal dose-response model in Prism. P values lower than 0.05 were considered statistically significant. All statistical tests were two-sided.

#### Sequencing and variant calling analyses

Variant detection was performed in two ways. We first used the CLC Genomics Workbench software v7.5 (Qiagen). We then used a dedicated amplicon analysis pipeline implemented in the Sequana v0.7.2 software^68^. The second method aligns the high-quality CCS reads on the reference sequence using the minimap2 aligner v2.8 software^69^ and calls for variants using the FreeBayes v1.2 software^70^. We focused on single nucleotide polymorphisms (SNPs) and small insertions and deletions (INDELs) and found a good agreement between the two methods. The patient CCR5 genotypes deduced from sequencing were compared to CCR5 reference haplotypes, as reported in Table 1. The SNPs and INDELs reported were highly supported, with a mean sequencing depth above 600 across the different patients.

### Reporting summary

Further information on research design is available in the Nature Research Reporting Summary linked to this article.

## Supplementary information

Supplementary information

Reporting Summary

## Data Availability

Source data are provided with this paper. Data corresponding to Figs. 1 to 8 and Supplementary Figs 1 to 10 are provided in the accompanying Source Data excel file. CCR5 sequences have been deposited to the European Nucleotide Archive (ENA) under the ArrayExpress accession code E-MTAB-11062. Source data are provided with this paper.

## Source data

Source Data

## Code availability

No code was generated for this study. Source data are provided with this paper.
