# Supplementary material for: Low CCR5 expression protects HIV-specific CD4+ T cells of elite controllers from viral entry
Source: Nat Commun. 2022 Jan 26;13:521. doi: 10.1038/s41467-022-28130-0 (PMC8792008; doi:10.1038/s41467-022-28130-0)
Supplement: Supplementary file 1 — Supplementary information [file 41467_2022_28130_MOESM1_ESM.pdf]

Low CCR5 expression protects HIV-specific CD4+ T cells of elite controllers from viral entry

Mathieu Claireaux*, Rémy Robinot*, Jérôme Kervevan**, Mandar Patgaonkar**, Isabelle Staropoli, Anne Brelot, Alexandre Nouël, Stacy Gellenoncourt, Xian Tang, Mélanie Héry, Stevenn Volant, Emeline Perthame, Véronique Avettand-Fenoël, Julian Buchrieser, Thomas Cokelaer, Christiane Bouchier, Laurence Ma, Faroudy Boufassa, Samia Hendou, Valentina Libri, Milena Hasan, David Zucman, Pierre de Truchis, Olivier Schwartz, Olivier Lambotte, and Lisa A. Chakrabarti***

* These authors contributed equally: M. Claireaux, R. Robinot
** These authors contributed equally: J. Kervevan, M. Patgaonkar
*** Correspondence: chakra@pasteur.fr

SUPPLEMENTARY INFORMATION CONTENT

Supplementary Figure 1: Strategy for the single cell analysis of Gag293-specific CD4+ T cells
Supplementary Figure 2: Comparisons of gene and protein expression profiles in Gag293-specific CD4+ T cells (Tet+) and non-specific memory CD4+ T cells (Tet-) in HIV controllers (HIC) and treated patients (ART)
Supplementary Figure 3: Distribution of single cell gene and protein expression
Supplementary Figure 4: CCR5 expression and fusion in CD4+ T cell subsets
Supplementary Figure 5: Longitudinal follow-up of Tetramer+ memory CD4+ T cells
Supplementary Figure 6: Quantification of CCR5 mutant expression
Supplementary Figure 7: Antigenic stimulation induce CCR5 downregulation in patient memory CD4+ T cells
Supplementary Figure 8: Effect of TCR stimulation on CCR5 expression
Supplementary Figure 9: measurement of β-chemokines in stimulated cultures and patient plasma
Supplementary Figure 10: CCR5 is dynamically internalized in CD4+ T cells upon Gag stimulation

Supplementary Table 1: Clinical characteristics and HLA-DR typing of patients included in the MHC II tetramer study
Supplementary Table 2: List of genes studied by single cell multiplexed real-time PCR
Supplementary Table 3: Antibody panel for single-cell sorting of Tet+ CD4+ T cells
Supplementary Table 4: Antibody panel for phenotyping total CD4+ T cells
Supplementary Table 5: Antibody panel for the analysis of HIV fusion in Tet+ CD4+ T cells
Supplementary Table 6: Antibody panel for the analysis of HIV fusion in total CD4+ T cells
Supplementary Table 7: Antibody panel for the analysis of HIV fusion in CCR5-nucleofected CD4+ T cells
Supplementary Table 8: Antibody panel for the analysis of CCR5 downregulation in patient CD4+ T cells
Supplementary Table 9: Antibody panel for the analysis of CCR5 expression after antibody feeding
Supplementary Table 10: Antibody panel for the analysis of TCR-dependent regulation of CCR5 expression in TCR-transduced PBMC

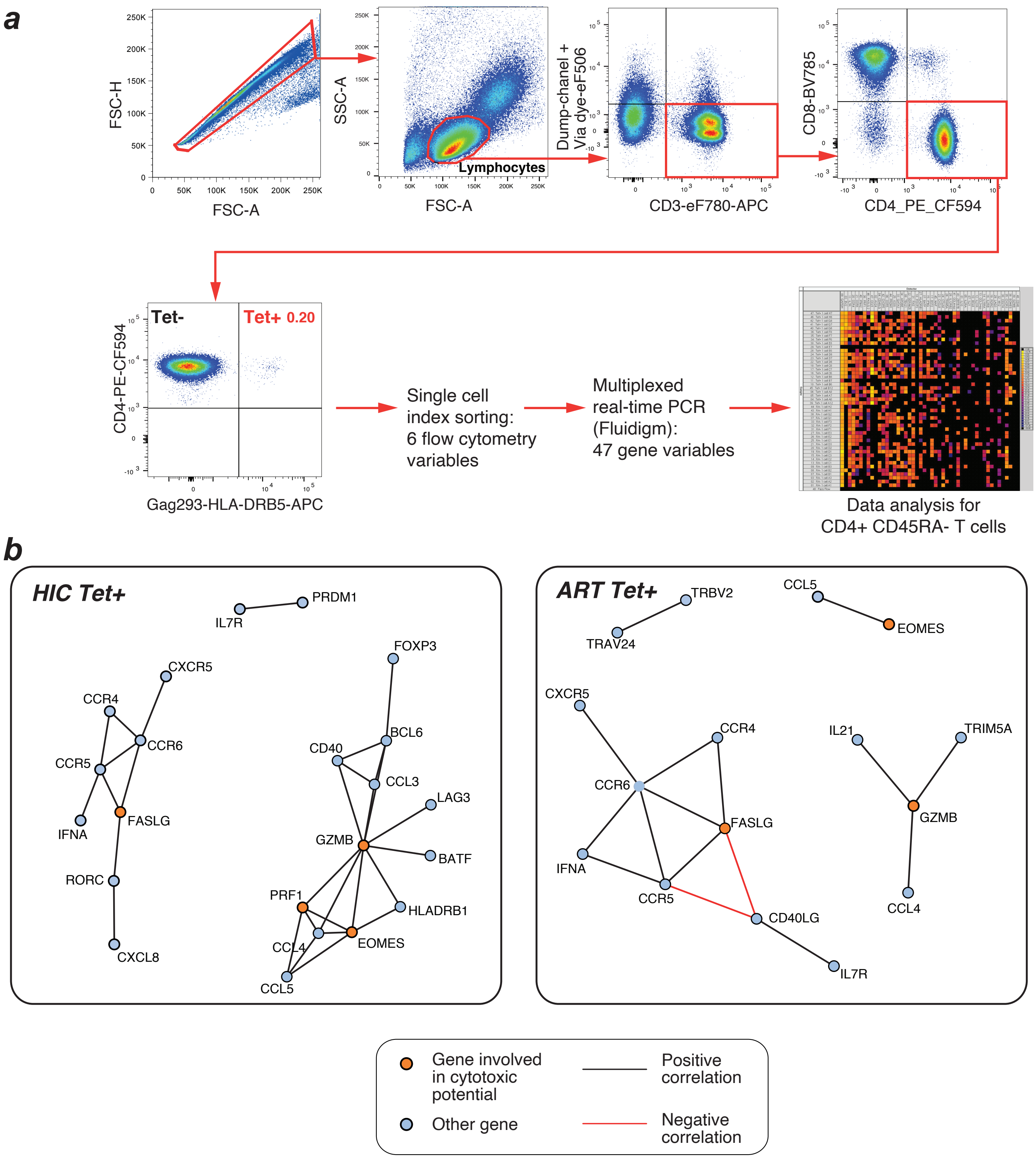

Supplementary Figure 1: Strategy for the single cell analysis of Gag293-specific CD4+ T cells

(a) Cell selection strategy: Gag293-specific CD4+ T cells (Tet+) and non-specific CD4+ T cells (Tet-) were isolated by indexed cell sorting in 96-well plates and analyzed by multiplexed real-time RT-PCR using a Biomark instrument (Fluidigm). Data analysis involved 6 flow cytometry and 47 gene variables derived from CD4+ CD45RA- single T cells. The analysis was carried out on cells from HIV controllers (HIC, n=9) and treated patients (ART, n=9), with 25 Tet+ cells and 25 Tet- cells analyzed for each patient tested.
(b) Network of co-expressed genes in Gag293-specific CD4+ T cells: gene communities which are positively (black lines) or negatively (red lines) correlated were inferred with the R «huge» and «mixer» packages based on Spearman correlation coefficients.

Claireaux et al.

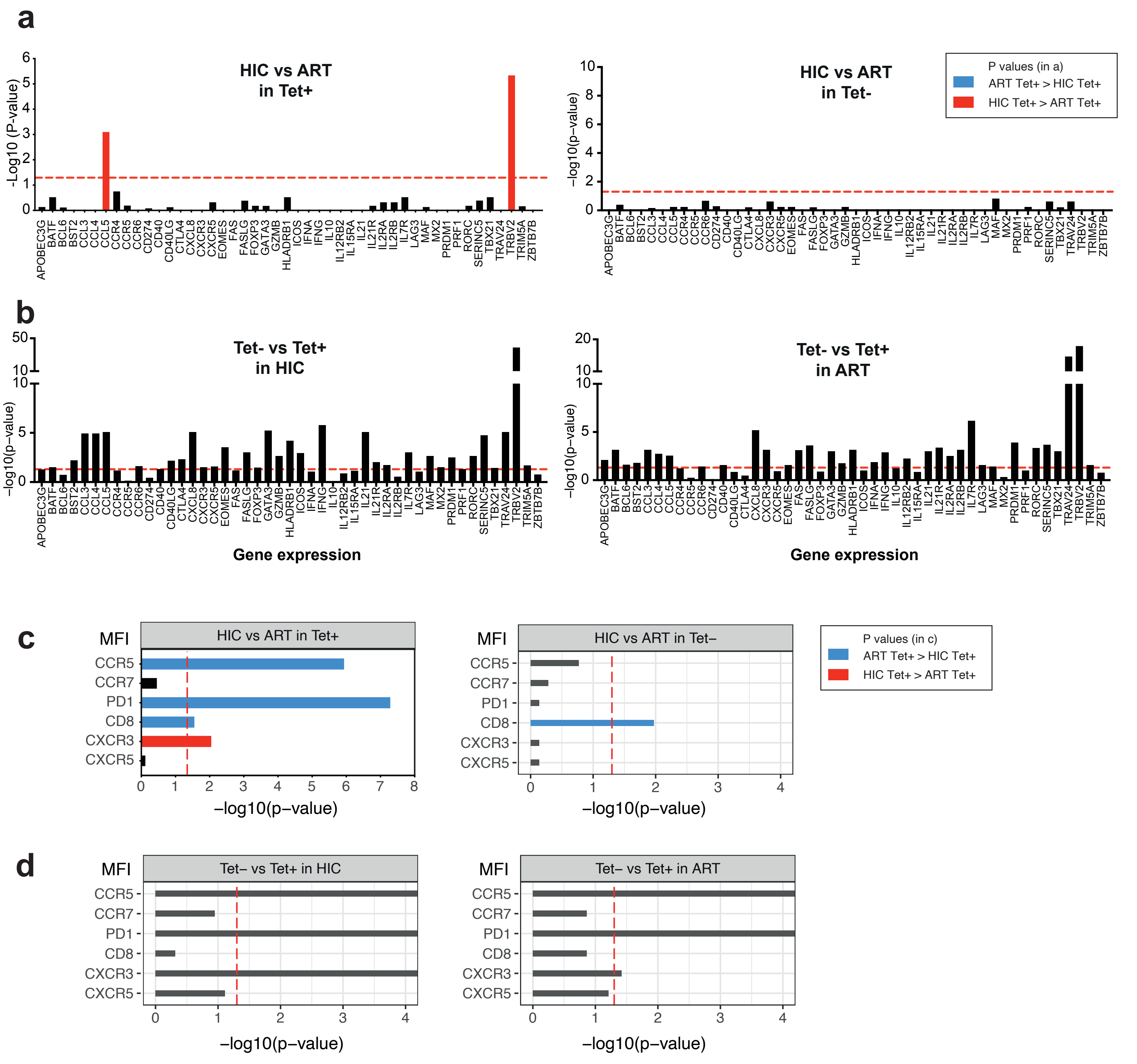

Supplementary Figure 2: Comparisons of gene and protein expression profiles in Gag293-specific CD4+ T cells (Tet+) and non-specific memory CD4+ T cells (Tet-) in HIV controllers (HIC) and treated patients (ART)

(a-d) Comparisons in were carried out for n=9 HIC and n=9ART patients.
P values obtained in the hurdle model and adjusted for multiple comparisons using the Benjamini and Hochberg procedure are reported. The significance threshold P=0.05 is indicated by a red dashed line.
(a) Comparisons of mRNA expression in HIC Tet+ vs ART Tet+ (left) and HIC Tet- vs ART Tet- (right).
(b) Comparisons of mRNA expression in Tet- vs Tet+ in HIC (left) and Tet- vs Tet+ in ART (right).
(c) Comparisons of protein surface expression in HIC Tet+ vs ART Tet+ (left) and HIC Tet- vs ART Tet- (right).
(d) Comparisons of protein surface expression in Tet- vs Tet+ in HIC (left) and Tet- vs Tet+ in ART (right).
In (d), -log10(p-values) ≥4.2 were set at 4.2.

Gene expression (normalized to GAPDH)

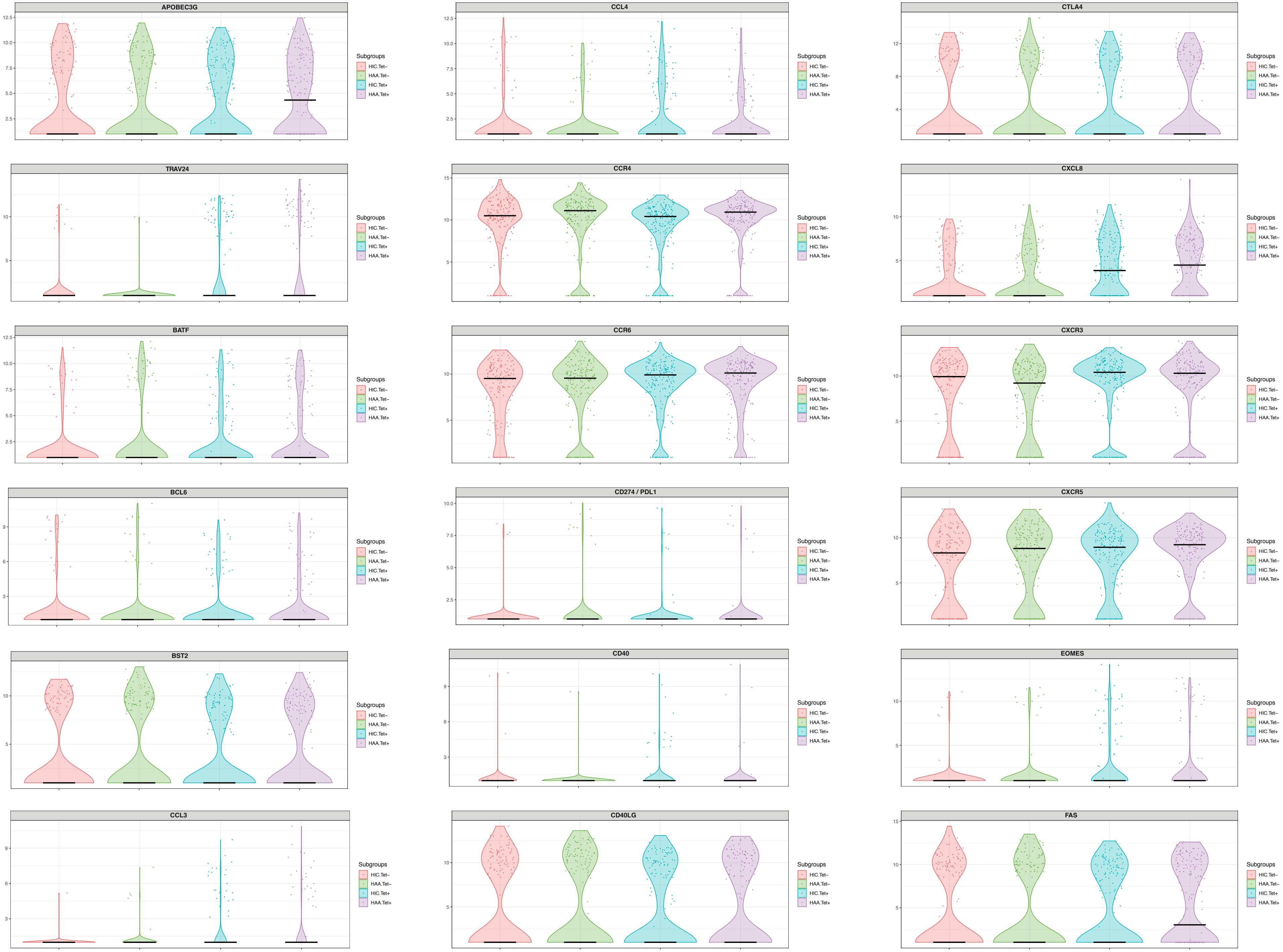

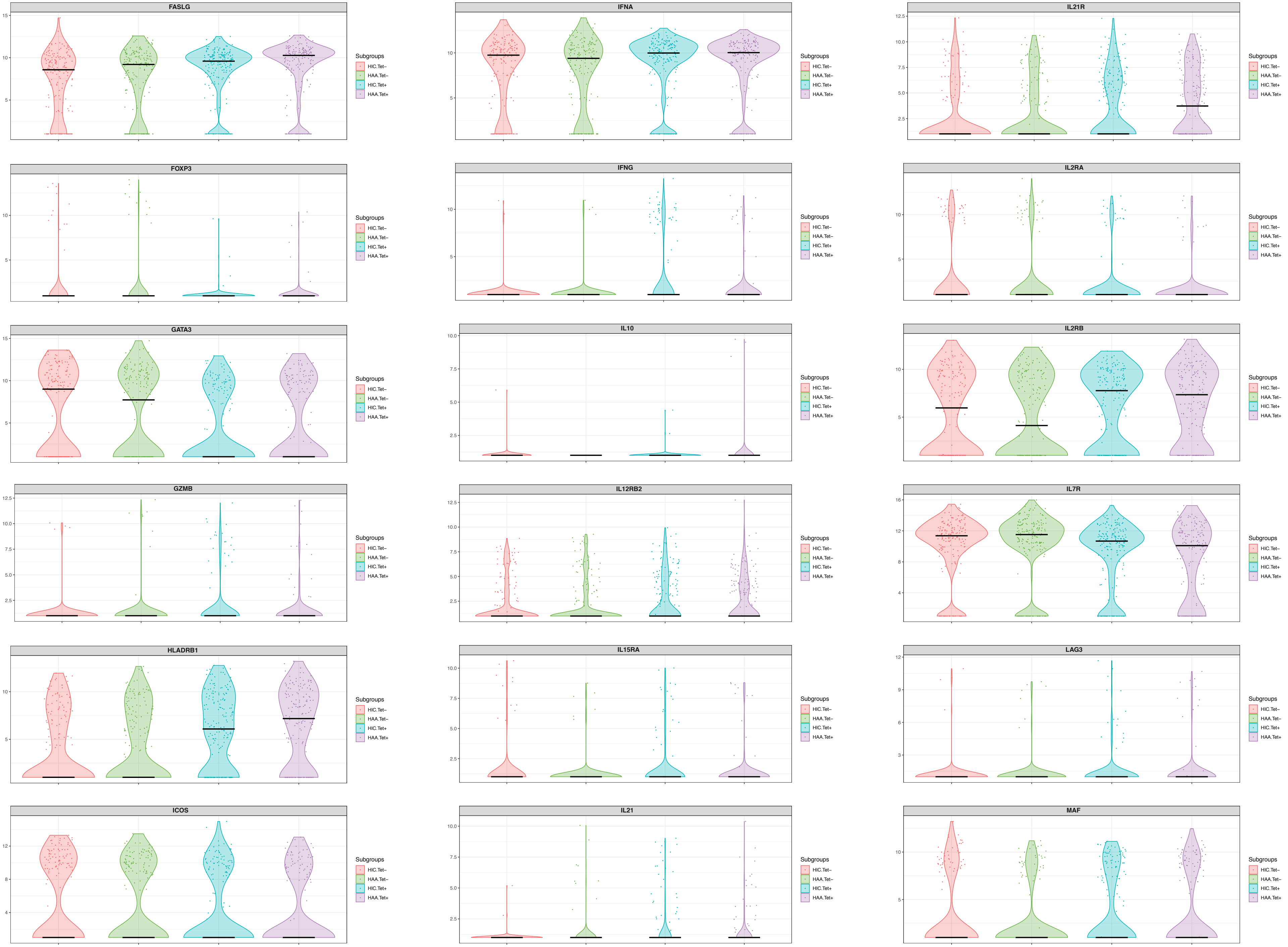
FASLG
IFNA
IL21R
FOXP3
IFNG
IL2RA
GATA3
IL10
IL2RB
GZMB
IL12RB2
IL7R
HLADRB1
IL15RA
LAG3
ICOS
IL21
MAF
Subgroups
HIC.Tet−
HAA.Tet−
HIC.Tet+
HAA.Tet+

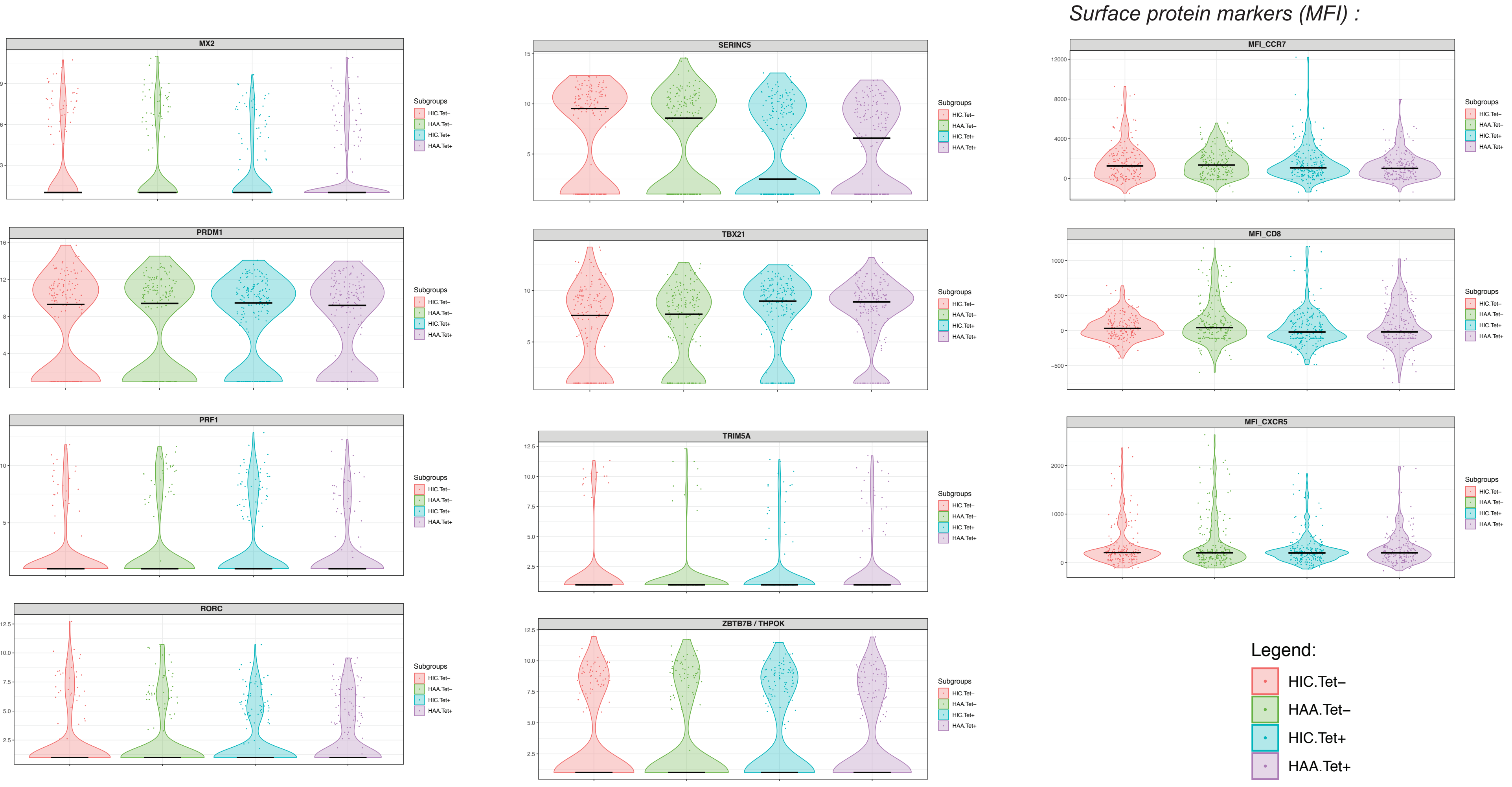

Supplementary Figure 3: Distribution of single cell gene and protein expression

The distribution of mRNA expression measured by multiplexed real time PCR (Fluidigm) and of surface protein expression measured by flow cytometry (MFI: mean fluorescence intensity) is reported for 700 single CD4+ T cells belonging to 4 groups: HIC Tet+ (n=188; cyan), ART Tet+ (n=170; purple), HIC Tet-(n=160; red), and ART Tet- (n=182; green). The black bars represent medians.

Claireaux et al.

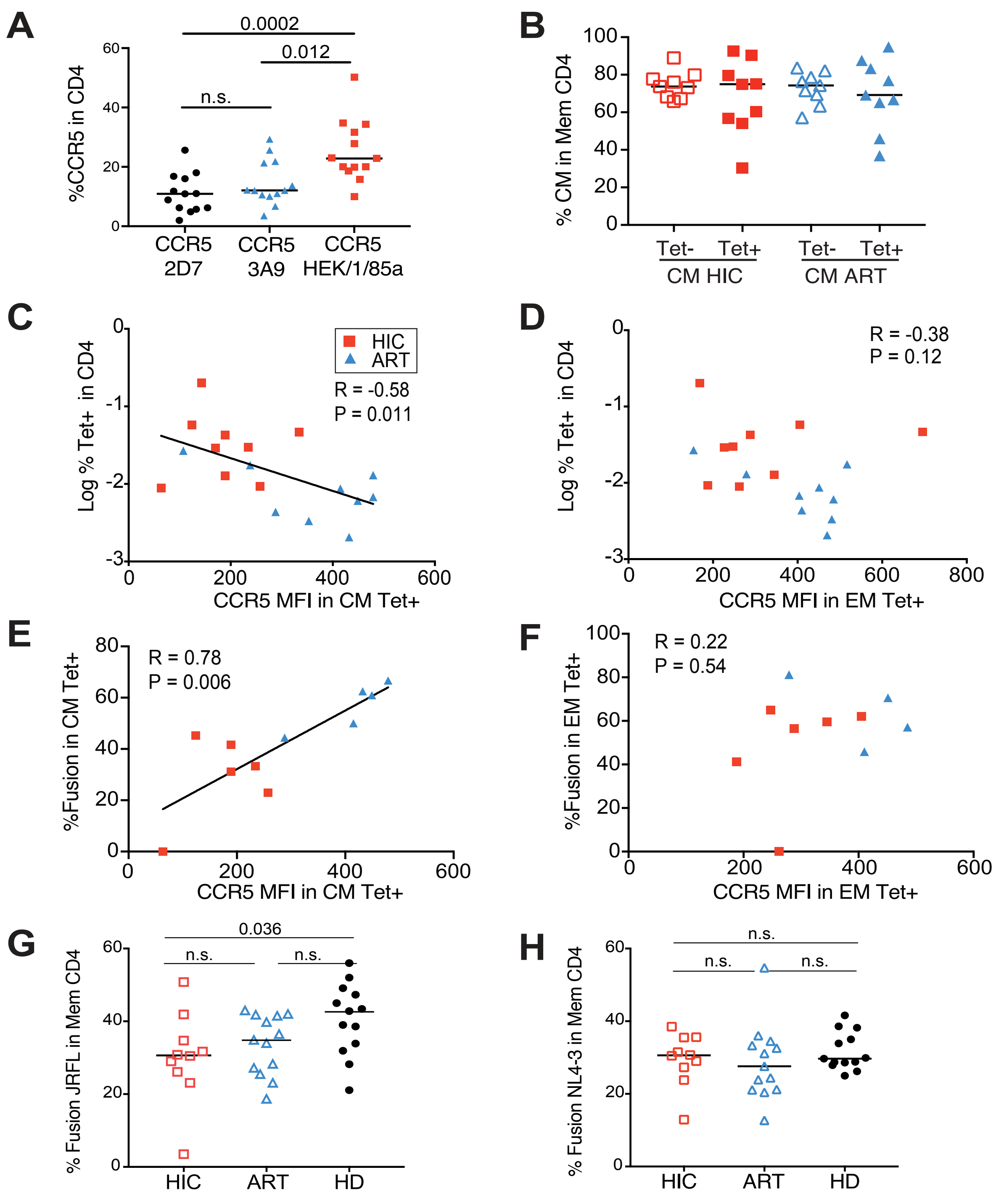

Supplementary Figure 4: CCR5 expression and fusion in CD4+ T cell subsets

(A) Comparison of CCR5 surface staining of CD4+ T cells from healthy donors (n=13) with three different antibodies.
(B) Frequency of central memory cells (CM) in Gag293-specific (Tet+) and non-specific (Tet-) memory CD4+ T cells of HIC (n=9) and ART (n=9) patients.
(C, D) Correlation between the frequency of Tet+ cells in CD4+ T cells and CCR5 expression in the subsets of CM Tet+ cells (C) and effector memory (EM) Tet+ cells (D). MFI: mean fluorescence intensity.
(E, F) Correlation between HIV-1 JR-FL fusion and CCR5 expression in the CM Tet+ (E) and EM Tet+ (F) populations.
(G, H) HIV fusion in the CD45RA- CD4+ T cell population (Mem CD4) of HIV controllers (HIC, n=10), treated patients (ART, n=13) and healthy donors (HD, n=13) after inoculation with HIV-1 JR-FL (G) or HIV-1 NL4-3 (H) virions.
(A-H) Horizontal bars represent medians; P values for differences between two categories were computed with the Mann-Whitney U test. Non parametric Spearman correlation coefficients (R) and associated P values are reported.

Claireaux et al.

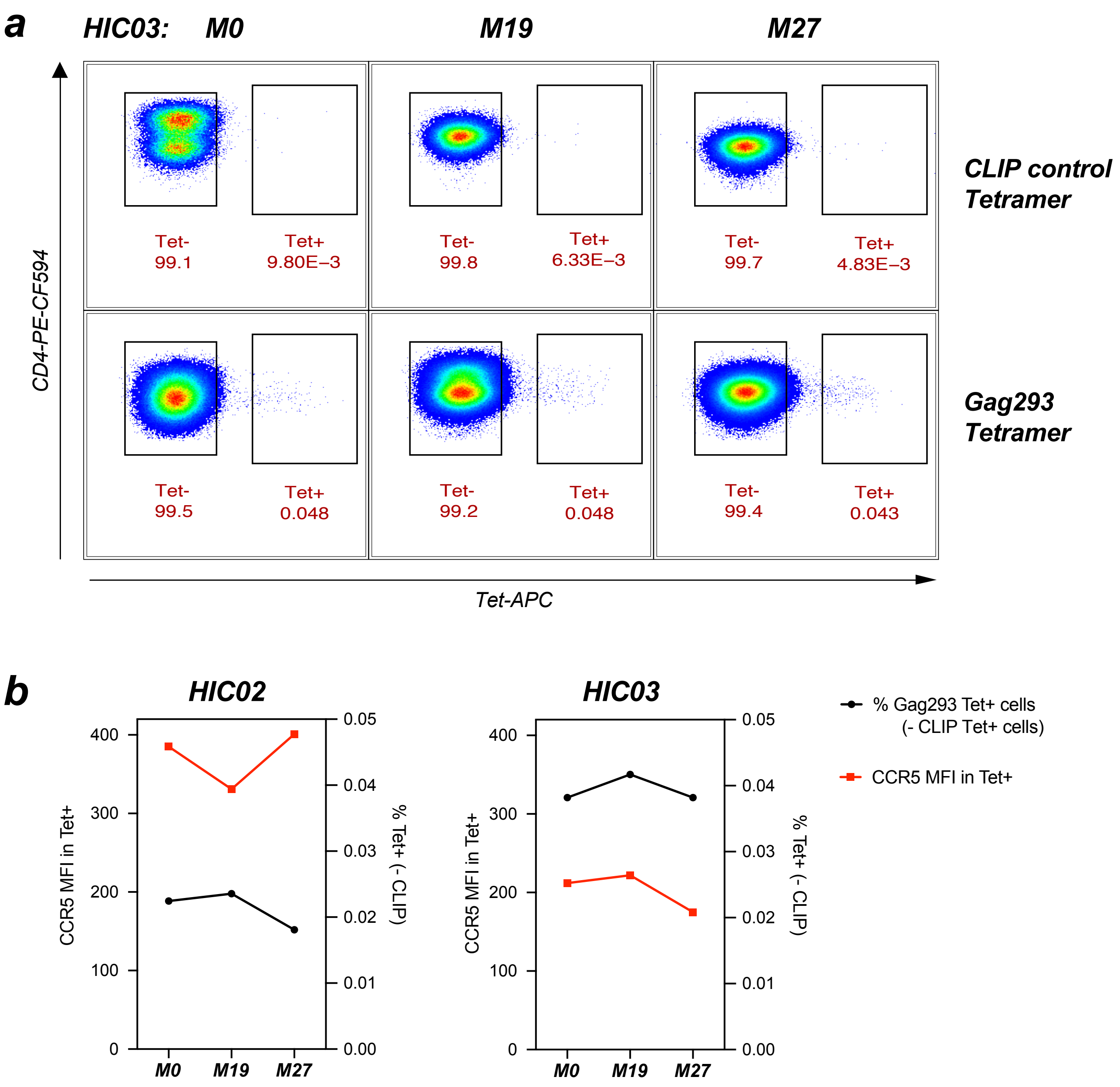

Supplementary Figure 5: Longitudinal follow-up of Tetramer+ memory CD4+ T cells

(a) Example of longitudinal analysis of Gag293-specific memory CD4+ T cells (Tet+) in a controller patient. MHC II tetramer labeling was performed with a control tetramer (CLIP, top row) and a specific tetramer (Gag293, bottom row) in CD4+ CD45A- viable T cells from sequential samples obtained at months M0, M19, and M27 from patient HIC03.

(b) The frequency of Gag293-specific Tet+ cells after CLIP background subtraction (black line) is reported over time for n=2 controller patients, HIC02 (left panel) and HIC03 (right panel). CCR5 expression levels in the Tet+ population are reported in the same panels by red lines. MFI: mean fluorescence intensity.

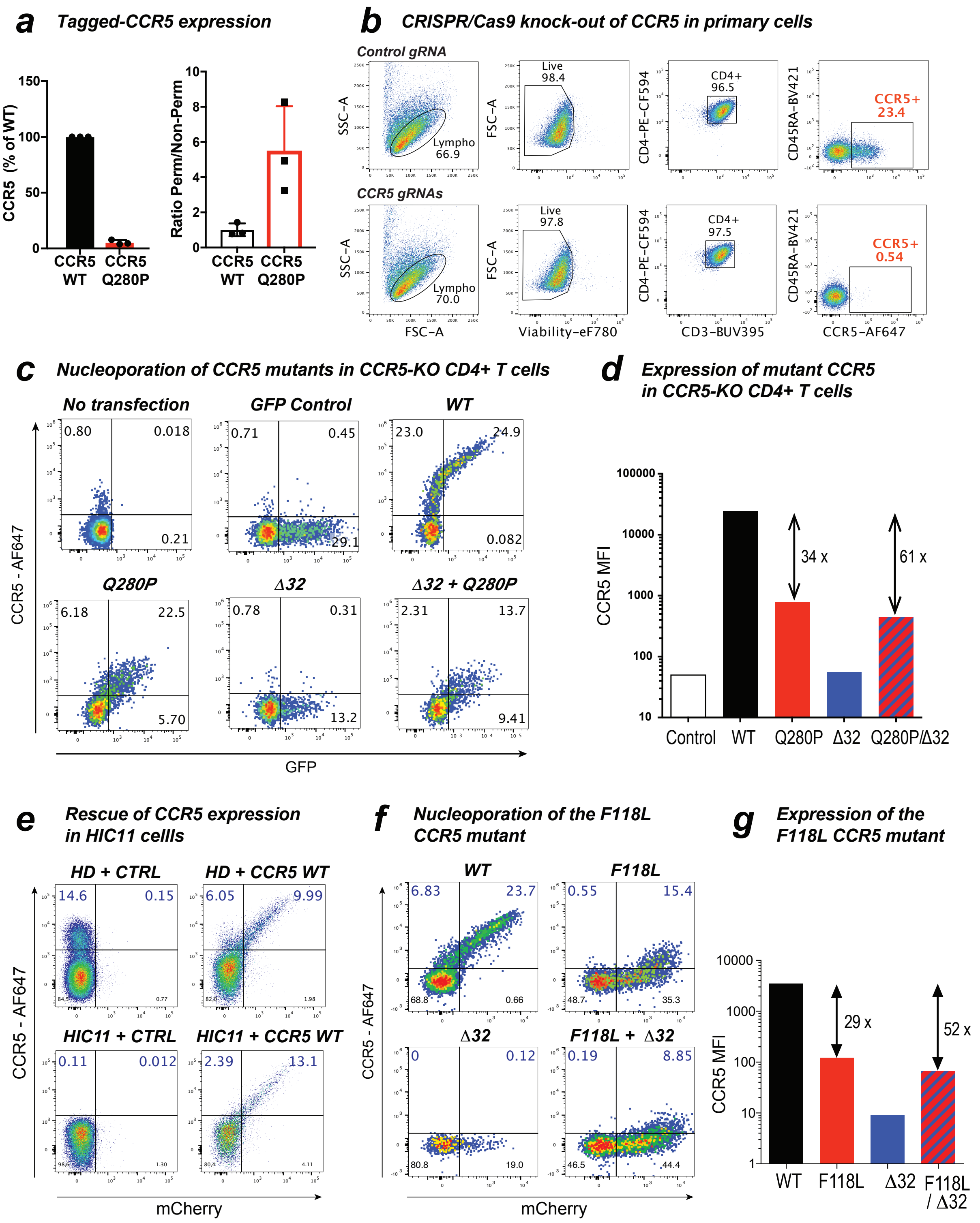

Supplementary Figure 6: Quantification of CCR5 mutant expression

(a) Left: Analysis of CCR5 surface expression in 293 cells transfected with FLAG-tagged WT and mutant CCR5 plasmids. Normalized MFI detected with an anti-FLAG antibody is reported. Right: ratio of FLAG labeling in saponin-permeabilized to non-permeabilized CCR5-FLAG expressing 293-T cells (n=3). Means ±SD are shown.
(b) Efficiency of CCR5 knock-out in primary CD4+ T cells nucleoporated with CRISPR/Cas9 RNP plus a control guide RNA (top row) or a mix of 3 CCR5 gRNAs (bottom row). (c) Nucleoporation of CCR5-T2A-GFP WT or mutant plasmids in CCR5 KO primary CD4+ T cells (n=1). (d) Quantification of CCR5 MFI in GFP+ CD4+ T cells for plots shown in (c).
(e) CCR5, expression is analyzed in total CD4+ T cells of one healthy donor (HD) and of patient HIC11, either untreated (CTRL) or nucleoporated with CCR5 WT plasmid.
(f) Nucleoporation of CCR5-T2A-mCherry WT or mutant plasmids in primary cells. Analysis is shown after gating in naive CD4+ T cells. (g) Quantification of CCR5 MFI in mCherry+ naive CD4+ T cells for plots shown in (F). One representative experiment out of n=2 is shown.

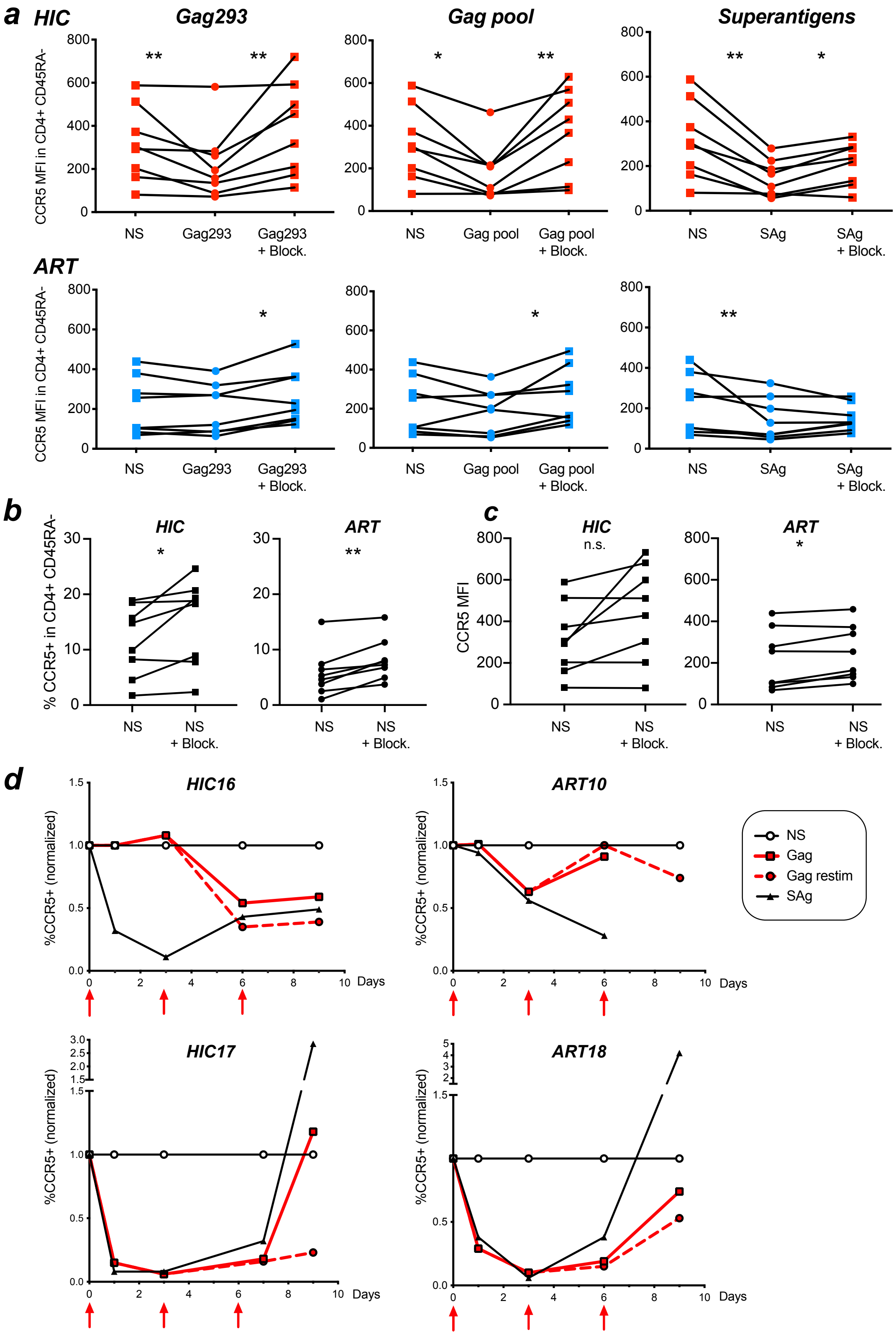

Supplementary Figure 7: Antigenic stimulation induce CCR5 downregulation in patient memory CD4+ T cells

(a) Quantitation of CCR5 mean fluorescence intensity (MFI) in memory CD4+ T cells of HIV controllers (HIC, top) and treated patients (ART, bottom) without stimulation (NS) or post-stimulation with the Gag293 peptide, a Gag peptide pool (Gag pool), or the superantigens SEA and SEE (SAg). Stimulations were done in the absence or presence (+ Block.) of blocking β-chemokine antibodies.
*(b, c) Analysis of the % of CCR5+ cells (b) and of the CCR5 MFI (c) in CD4+ CD45RA- memory T cells in the absence of antigenic stimulation (NS) shows that the addition of β-chemokine antibodies (+Block.) is sufficient to increase CCR5 expression, pointing to a degree of continuous CCR5 internalization mediated by chemokines, even at baseline. Variations in %CCR5+ cells (b) and in CCR5 MFI (a, c) within CD4+ CD45RA- memory T cells were analyzed in n=8 HIC and n=8 ART patients with the Wilcoxon matched-pairs signed rank test: n.s. not significant; * $P<0.05$., ** $P<0.01$.*
(d) Kinetics of CCR5 downregulation upon stimulation with a pool of Gag peptides (Gag pool) or superantigens (sAg). CCR5 expression in the CD45RA- CD4+ T cell population was monitored for 10 days post-stimulation in cells from n=2 controllers (left panels) and n=2 treated patients (right panels). The proportion of CCR5+ cells as compared to the unstimulated condition (NS) is reported. In the "Gag pool restim" condition, cultures were restimulated at days 3 and 6 with the same Gag peptide pool, as indicated by red vertical arrows.

Claireaux et al.

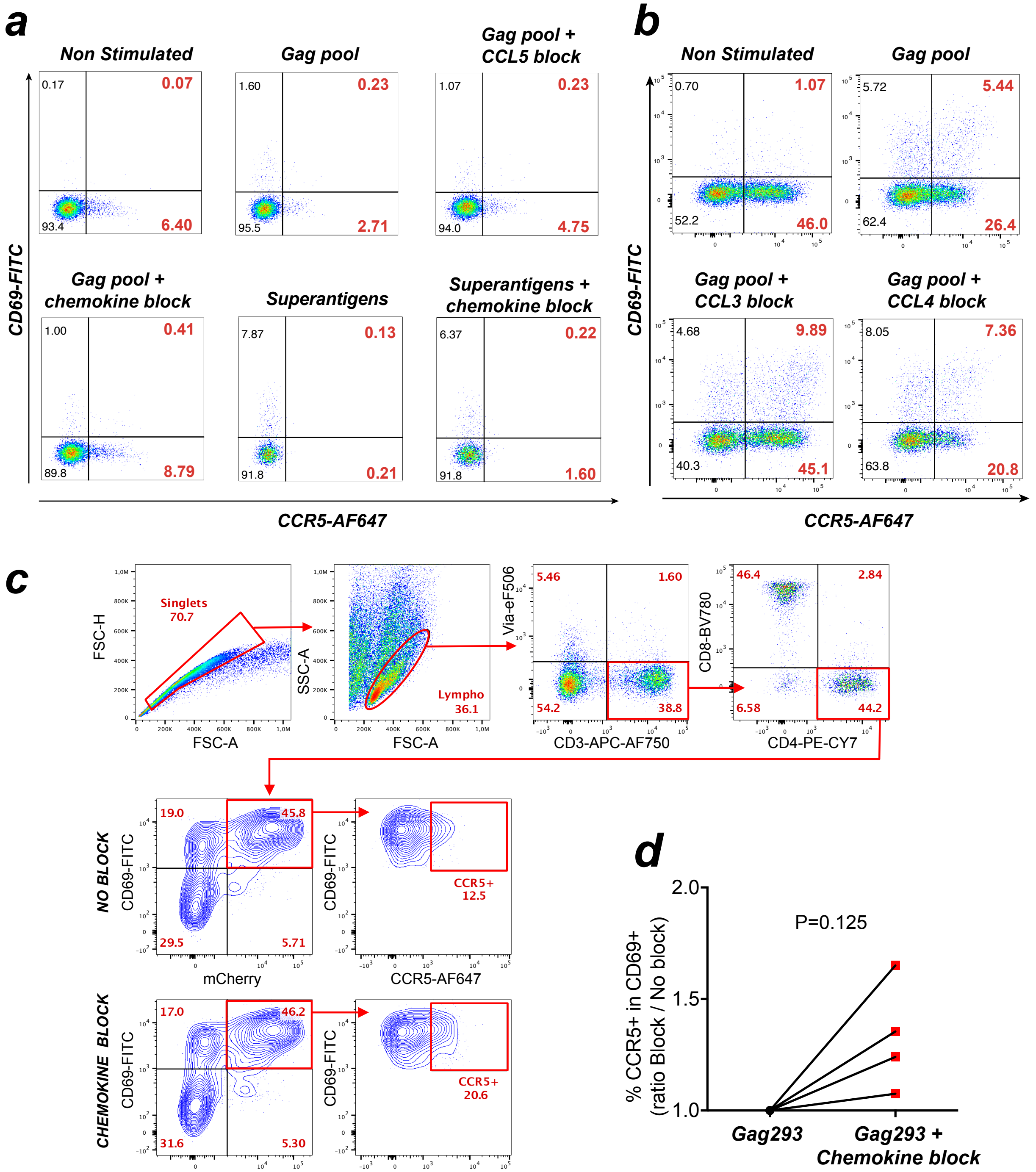

Supplementary Figure 8: Effect of TCR stimulation on CCR5 expression

(a) Example of CCR5 downregulation upon TCR stimulation in a treated patient. CCR5 expression was measured in the CD4+ CD45RA- T cell population after 3 days of stimulation with a Gag peptide pool. Incubation with a CCR5 blocking antibody partially inhibited CCR5 downregulation (top right), while a cocktail of 3 chemokine blocking antibodies to CCL3, CCL4, and CCL5 abrogated CCR5 downregulation (bottom left). Superantigen stimulation caused complete CCR5 downregulation, that was only partially reverted by chemokine blocking (bottom middle and right).
(b) Example of CCR5 downregulation upon TCR stimulation in a controller patient. A blocking antibody to CCL3 inhibited CCR5 downregulation (bottom left), while a blocking antibody to CCL4 did not (bottom right).
(c) Gating strategy to measure CCR5 downregulation in CD4+ T cells transduced with the F24 TCR. Cells were stimulated overnight with Gag293-pulsed antigen-presenting cells in the presence or absence of chemokine blocking antibodies. CCR5 expression was and analyzed in the TCR transduced (mCherry+) activated (CD69+) CD4+ T cell population.
(d) F24-transduced CD4+ T cells show partial inhibition of CCR5 downregulation upon stimulation with Gag293 in the presence of chemokine blocking antibodies. The ratio of the % CCR5+ cells without block to the % CCR5+ cells with block is reported for mCherry+ CD69+ CD4+ T cells. P value were computed with the Wilcoxon matched pairs signed rank test (n=4).

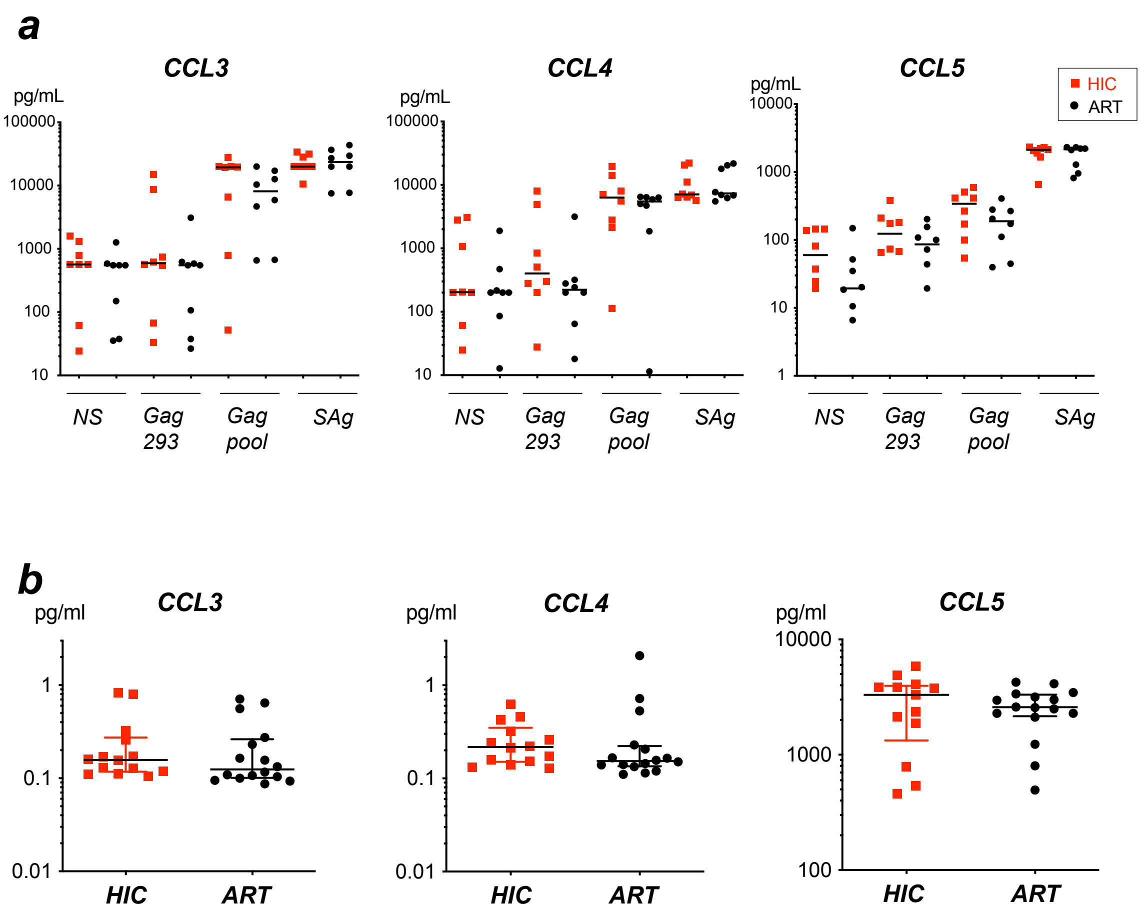

Supplementary Figure 9: measurement of β-chemokines in stimulated cultures and patient plasma

(a) The secretion of the chemokines CCL3 (left panel), CCL4 (middle panel) and CCL5 (right panel) was measured by ELISA assay in supernatants of patient PBMC cultures after 3 days of antigenic stimulation. Cultures were either not stimulated (NS) or were stimulated with the sole Gag293 peptide (Gag 293), with a pool of Gag peptides (Gag pool), or with the superantigens SEA and SEE (SAg). Comparisons of chemokine levels between HIV controllers (HIC, n=8) and treated patients (ART, n=8) were not statistically significant (P≥0.05) for all conditions tested, as determined by the Mann Whitney test. Bars represent median values.

(b) Concentrations of the chemokines CCL3 (left panel), CCL4 (middle panel) and CCL5 (right panel) were measured by ELISA assay in the plasma of HIC (n=14 for CCL3 and CCL4; n=13 for CCL5) and ART (n=16) patients. Comparisons of chemokine levels between the two patient groups were not statistically significant (P≥0.05) for all chemokines tested, as determined by the Mann Whitney test. Bars represent medians with interquartilel range.

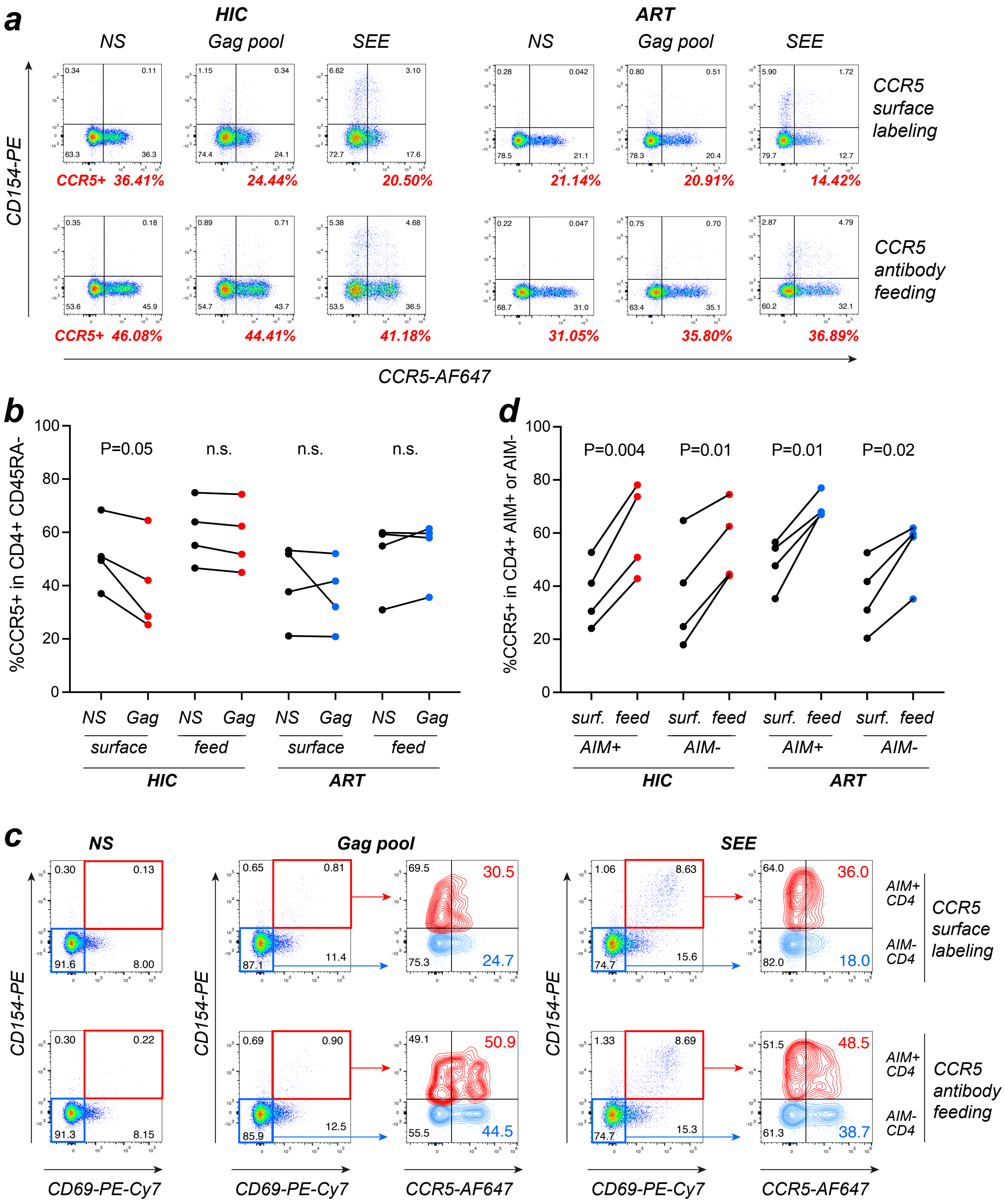

Supplementary Figure 10: CCR5 is dynamically internalized in CD4+ T cells upon Gag stimulation

(a) CCR5 expression measured by surface antibody labeling (top row) and antibody feeding (bottom row) in memory CD4+ T cells of two representative patients, one HIV Controller (HIC) and one treated patient (ART). Memory CD4+ T cells gated as singlet viable CD4+ CD4+ CD45RA- T cells were analyzed for CD154 and CCR5 expression 18h post stimulation in control unstimulated conditions (NS) or after stimulation with a Gag peptide pool (Gag pool) or a superantigen (SEE).

(b) Analysis of CCR5 expression measured by surface antibody labeling (surface) and antibody feeding (feed) in memory CD4+ T cells left unstimulated (NS) or stimulated with a Gag peptide pool (Gag) in n=4 patients from the HIC and ART groups, respectively. P values estimating the decrease of CCR5 upon stimulation were computed with a paired t test.

(c) CCR5 expression measured by surface antibody labeling (top row) and antibody feeding (bottom row) in Gag-specific (AIM+ CD4: CD154+ CD69+) and non-specific (AIM-: CD154- CD69-) memory CD4+ T cells stimulated with a Gag peptide pool (Gag pool) or a superantigen (SEE) for 18h in an activation marker-induced (AIM) assay, for one representative HIC patient.

(d) Analysis CCR5 expression measured by surface antibody labeling (surf.) and antibody feeding (feed) in AIM+ and AIM- memory CD4+ T cells stimulated with a Gag peptide pool in n=4 patients from the HIC and ART groups, respectively. P values estimating the increase of CCR5 detection upon antibody feeding were computed with a paired t test.

A

Patient group	Duration of infection years	Viral load HIV-1 copies/mL	CD4 /mm3	Nadir of CD4+ T cells /mm3	Duration of antiretroviral treatment	Age
**HIC**	17.6 [8.3 - 27.4]	<50	972 [315 - 1333]	N/A	N/A	50.7
**ART**	17.7 [8.9 - 27.2]	<50	427 [211 - 987]	172 [10 - 356]	9.5 [6.4 - 20.0]	49.9
**P value* HIC vs ART**	N.S.	N.S.	P=0.004	-	-	N.S.

B

Patient ID	Duration of infection years	Viral load HIV-1 copies/mL	CD4 /mm3	HLA-DR typing	HLA-DR tetramer used	Ultrasensitive Viral load HIV-1 copies/mL
**HIC01**	11.6	<50	744	DR7, DR15, DRB5	DRB5	<1.2
**HIC02**	23.9	<50	1333	DR1, DR11	DRB5	<3
**HIC03**	16.3	<50	944	DR11, DR12	DR11	<2
**HIC04**	9.0	<50	1252	DR14, DR16, DRB5	DRB5	6.8
**HIC05**	20.0	<50	315	DR1, DR7	DR7	<2
**HIC06**	26.0	<50	852	DR15, DRB5	DRB5	60
**HIC07**	18.9	<50	702	DR1, DR16	DR1	<2
**HIC08**	27.4	<50	1030	DR15, DRB5	DRB5	<1
**HIC09**	27.1	<50	1000	DR11, DR15, DRB5	DR11	<2
**HIC10**	10.8	<50	1016	DR8, DR13-2	DR13-2	<2
**HIC11**	8.3	<50	755	DR13, DR15, DRB5	DRB5	<2
**HIC12**	14.9	<50	1063	DR1, DR7	DR1	6
Median	17.6	<50	972			<2

C

Patient ID	Duration of infection years	Viral load HIV-1 copies/mL	CD4 /mm3	HLA-DR typing	HLA-DR tetramer used
**ART01**	19.0	<50	373	DR4, DR8	DR4
**ART02**	9.0	<50	427	DR1, DR3	DR1
**ART03**	19.0	<50	411	DR3, DR4	DR4
**ART04**	18.9	<50	328	DR1, DR13	DR1
**ART05**	8.9	<50	424	DR1, DR15, DRB5	DR1
**ART06**	11.9	<50	417	DR15, DR16, DRB5	DRB5
**ART07**	27.2	<50	615	DR1, DR7	DR1
**ART08**	13.7	<50	211	DR15, DR16, DRB5	DRB5
**ART09**	25.9	<50	403	DR4-5, DR7	DR4-5
**ART10**	12.3	<50	987	DR1, DR1	DR1
**ART11**	12.8	<50	754	DR9, DR16, DRB5	DRB5
**ART12**	20.8	<50	847	DR15, DRB5	DRB5
**ART13**	17.7	<50	639	DR7, DR11	DR11
**ART14**	9.7	<50	948	DR1, DR14	DR1
**ART15**	19.0	<50	784	DR7, DR11	DR11
Median	17.7	<50	427		

Supplementary Table 1: Clinical characteristics and HLA-DR typing of patients included in the MHC II tetramer study

12 HIV controllers (HIC) and 15 treated patient (ART) were included in the MHC II tetramer study.
*(A) Summary of clinical characteristics of studied patients. Median values and ranges are reported. * P values estimated with the Mann-Whitney U test are reported. N.S.: not significant. N/A: not available.*
(B, C) Clinical characteristics and HLA-DR typing of HIC (B) and ART (C) patients.
*Patients included in the study were genotyped for HLA-DRB1, and then analyzed with HLA-DR-matched MHC II tetramers loaded with the Gag293 peptide. The tetramers used for this analysis is reported in the rightmost column. The following tetramers were used: HLA DRB1*0101 (DR1),DRB1*0401 (DR4), DRB1*0405 (DR4-5), DBRB1*0701 (DR7), DRB1*1101 (DR11), DRB1*1302 (DR13-2), DRB1*1502 (DR15), and DRB5*0101 (DRB5).*

	Gene Symbol	Alternate symbol	Gene Name	NCBI Ref Seq	Function	Forward Primer	Reverse Primer	Amplicon (bp)
1	APOBEC3G		apolipoprotein B mRNA editing enzyme, catalytic polypeptide-like 3G	NM_021822.3	Restriction factor	CAACCAGGCTCCACATAAAC	AAGCAGGTAACCCTGTAGTC	117
2	HUTRAV24		human T cell receptor alpha variable 24	AE000660 (IMGT)	TCR	GTGAATAGGCAGACAGACTTGT	CCGAGGCCTTGTTTGTAATG	280
3	BATF		basic leucine zipper transcription factor; ATF-like	NM_006399.3	Tfh differentiation	CTGGCAAACAGGACTCATCT	CCTTCTGTGTCTGCCTCTG	99
4	BCL6		B-cell CLL/lymphoma 6	NM_001706.4	Tfh differentiation	TCGTTAACAGGTCCATGACG	GTGTGGAGGCACATCTCTG	130
5	BST2	Tetherin	bone marrow stromal antigen 2	NM_004335.3	Restriction factor	AGAGAAGGCCCAAGGACAA	TTTCTCTTCTCAGTCGCTCCA	110
6	HUTRBV2		human T cell receptor beta variable 2	L36092 (IMGT)	TCR	GCCAGGCAYACCAGTGT	GATCCGGTCCACAAAGCTGG	210
7	CCL3	MIP1a	chemokine (C-C motif) ligand 3	NM_002983.2	Th1 Diff./ inflammation	ATCATGCAGGTCTCCACT	GGAGGTGTAGCTGAAGCAG	120
8	CCL4	MIP1b	chemokine (C-C motif) ligand 4	NM_002984.3	Th1 Diff./ inflammation	GCACCAATGGGCTCAGAC	GGCTGCTGGTCTCATAGTAATC	100
9	CCL5	RANTES	chemokine (C-C motif) ligand 5	NM_002985.2	Th1 Diff./ inflammation	GAGTATTTCTACACCAGTGGCAAG	TCCCGAACCCATTTCTTCTCT	104
10	CCR4	CD194	chemokine (C-C motif) receptor 4	NM_005508.4	Other Th Differentiation	GACTGATGTGTACCTGCTCAAC	AATCATCTTGCACAGACCTAGC	121
11	CCR5	CD195	chemokine (C-C motif) receptor 5	NM_000579.3	Th1 Differentiation	ACTGACATCTACCTGCTCAAC	CACATTGTATTTCCAAAGTCCCA	110
12	CCR6	CD196	chemokine (C-C motif) receptor 6	NM_004367.5	Other Th Differentiation	CCTGAACCCTGTGCTCTAC	CTGAGGACTTGTACTTCCTTCTC	104
13	CD274	PDL1	Programmed death-ligand 1	NM_014143.3	Tfh differentiation	TGAATTGGTCATCCCAGAACTA	AGTGCTACACCAAGGCATAA	99
14	CD40	TNFRSF5	CD40 molecule, TNF receptor superfamily member 5	NM_001250.5	Activation	GCTGTCCATCCAGAACCAC	CCAGTTTCTGTCCTGGCT	94
15	CD40LG	CD154	CD40 ligand	NM_000074.2	Activation	GGTGATCAGAATCCTCAAATTGC	TGTTGCTCATGGTGTAGTATCC	106
16	CTLA4	CD152	cytotoxic T-lymphocyte-associated protein 4	NM_005214.4	Activation	CCCTGTCTTCTGCAAAGCAAT	CACTGTCACCCGGACCT	127
17	CXCL8	IL8	chemokine (C-X-C motif) ligand 8	NM_000584.3	Inflammation	ATAAAGACATACTCCAAACCTTTCCAC	AAGCTTTACAATAATTTCTGTGTTGGC	102
18	CXCR3	MIGR	chemokine (C-X-C motif) receptor 3	NM_001504.1	Th1 Differentiation	CAGGTGCCCTCTTCAACATC	GGTAGAGCTGGGTGGCAT	102
19	CXCR5	CD185	chemokine (C-X-C motif) receptor 5	NM_001716.4	Tfh differentiation	CCTGCACAAAGTCAACTTCTAC	CAGGTGATGTGGATGGAGAG	129
20	EOMES	TRB2	Eomesodermin	NM_001278182.1	Cytotoxicity	ACAACAACACCCAGATGATAGTC	TCTTTGAGGGCTCATTCAAGTC	111
21	FAS	CD95	Fas (TNF superfamily; member 6)	NM_000043.4	Apoptosis	GGACATGGCTTAGAAGTGGAA	GTTCACATTTGGTGCAAGGG	124
22	FASLG	CD95LG	Fas ligand (TNF superfamily; member 6)	NM_000639.2	Apoptosis	TTAACAGGCAAGTCCAACTCA	CCACCCTTCTTATACTTCACTCC	95
23	FOXP3		forkhead box P3	NM_014009.3	Other Th Differentiation	CCTCCAGAGAGAGATGGTACAG	CGGATGATGCCACAGATGAA	128
24	GATA3		GATA binding protein 3	NM_001002295.1	Other Th Differentiation	TGGAGGAGGAATGCCAATG	TTCGGTTTCTGGTCTGGATG	118
25	GZMB	CTLA1	granzyme B (granzyme 2)	NM_004131.4	Cytotoxicity	GCAACCAATCCTGCTTCTG	TCCCAGATCATAAGATAAGCCAT	123
26	HLA-DRB		HLA class II histocompatibility antigen, DR beta chain	NM_002124.3	Activation	CCTTCCAGACCCTGGTGAT	TGCTCTCCATTCCACTGTGA	110
27	ICOS	CD278	inducible T-cell co-stimulator	NM_012092.3	Tfh differentiation	TGTGCAGCCTTTGTTGTAGT	GCTGTGTTCACTGCTCTCAT	128
28	IFNA2		interferon; alpha 2	NM_000605.3	Inflammation	GCATCTGCAACATCTACAATGG	CAGATCACAGCCCACAGAG	96
29	IFNG		interferon; gamma	NM_000619.2	Th1 Differentiation	AGTGTGGAGACCATCAAGGA	GGACATTCAAGTCAGTTACCGAA	112
30	IL10		interleukin 10	NM_000572.2	Other Th Differentiation	GAGATGCCTTCAGCAGAGT	ACCCAGGTAACCCTTAAAGTC	104
31	IL12RB2		interleukin-12 receptor; beta 2	NM_001559.2	Th1 Differentiation	TTCTGGAAGAATCTGAGTGTCTC	GATGTTCTGTGTCATGGCTTTC	102
32	IL15RA	CD215	interleukin-15 receptor; alpha	NM_001256765.1	Activation	CTCTCAGACAACAGCCAAGA	GGAGATAGCCACAGTGGTG	103
33	IL21		interleukin-21	NM_021803.3	Tfh differentiation	AAGGTCAAGATCGCCACAT	AGTTTGTCTCTACATCTTCTGGA	123
34	IL21R	CD360	interleukin-21 receptor	NM_181078.2	Tfh differentiation	TCAGAGGAGTTAAAGGAAGGCT	CCACAATGGATGGGTCTTCAG	105
35	IL2RA	CD25	interleukin-2 receptor; alpha	NM_000417.2	Other Th Differentiation	AAAGCGGGTCACTCTATATGC	GGTGTCACTTGTTTCGTTGTG	112
36	IL2RB	CD122	interleukin-2 receptor; beta	NM_000878.3	Activation	CGTCCAGAAGTGGCTCTC	TCACCTTGTCCCTCTCCA	104
37	IL7R	CD127	interleukin-7 receptor	NM_002185.3	Activation (anti)	TTTAATGCACGATGTAGCTTACC	CCGGTTGGAGCTTTCTCT	107
38	LAG3	CD223	lymphocyte-activation-gene-3	NM_002286.5	Activation	CGACTTTACCCTTCGACTAGAG	TGGGAGTCACTGTGATGATTG	125
39	MAF		v-maf musculoaponeurotic fibrosarcoma oncogene homolog (avian)	NM_005360.4	Tfh differentiation	AGGAGAAATACGAGAAGTTGGT	TGGCGTATCCCACTGATG	129
40	MX2	MxB	Interferon-induced GTP-binding protein Mx2	NM_002463.1	Restriction factor	GGACAGGACCATCGGTATC	TCACAATCATGTAGCCCTTCT	119
41	PRDM1	Blimp1	PR domain containing 1; with ZNF domain	NM_001198.3	Th1 Differentiation	CCAGGAACTTCTTGTGTGGTA	GCTCTGTGTTTGTGTGAGATTC	100
42	PRF1		perforin 1 (pore forming protein)	NM_005041.4	Cytotoxicity	CCGCTTCTACAGTTTCCATGTG	AGTTGGAGATAAGCCTGAGGTAG	128
43	RORC		RAR-related orphan receptor C	NM_005060.3	Other Th Differentiation	ATCAGCTCCATCTTTGACTTCT	CCGATGGGCATTGATGAGA	102
44	SERINC5		serin incorporator 5	NM_001174072.2	Restriction factor	ATCGAGTTCTGACGCTCTGC	GCTCTTCAGTGTCCTCTCCAC	101
45	TBX21	TBET	T-box 21	NM_013351.1	Th1 Differentiation	GGCTGCATATCGTTGAGGT	GAGTAATCTCGGCATTCTGGTAG	129
46	TRIM5a	RNF88	Tripartite motif-containing protein 5	NM_033034.2	Restriction factor	GACGCTACTGGGTTGATGTG	CGGAGAGCTCACTTGTCTCTTA	123
47	ZBTB7B	THPOK	zinc finger and BTB domain containing 7B	NM_001252406.2	Cytotoxicity (anti)	CTGTCTGCCACAAGATCATCC	GCTTGTCGTTCCTGGTGAAT	120
48	GAPDH		glyceraldehyde-3-phosphate dehydrogenase	NM_002046.5	Housekeeping gene	GATCATCAGCAATGCCTCCT	GACTGTGGTCATGAGTCCTTC	100

Supplementary Table 2: List of genes studied by single cell multiplexed real-time PCR

Genes belonging to the same functional group are labeled with the same color in the "Function" column.

Claireaux et al.

Supplementary Table 3: Antibody panel for single-cell sorting of Tet+ CD4+ T cells

Marker	*fluorochrome*	*Manufacturer*	*Clone*	*Dilution*
CD3	eF780-APC	eBioscience	UCHT1	1:100
TCR or Tetramer	APC	eBioscience/-	IP26 / -	1:100/-
Viability Dye	eFluor 506	eBioscience	-	1:200
CD4	PE-CF594	BD Biosciences	RPA-T4	1:200
CXCR5	AF488	BD Biosciences	RF8B2	3:100
CXCR3	BV605	BD Biosciences	1C6/CXCR3	3:100
CD14	Viogreen	Miltenyi Biotec	TÜK4	3:100
CD20	Viogreen	Miltenyi Biotec	LT29	3:100
CD8	BV785	Biolegend	RPA-T8	1:200
CD45RA	BV421	Biolegend	HI100	1:100
CCR7	PE-Cy7	Biolegend	G043H7	2:100
CCR5	PerCP-Cy5-5	Biolegend	HEK/1/85a	3:100

Supplementary Table 4: Antibody panel for phenotyping total CD4+ T cells

Marker	*fluorochrome*	*Manufacturer*	*Clone*	*Dilution*
Viability Dye	eFluor 780	eBioscience	-	1:200
CD4	PE-CF594	BD Biosciences	RPA-T4	1:200
CD45RA	BV421	Biolegend	HI100	1:100
CD3	BUV395	BD Biosciences	SK7	3:100
HLA-DR	FITC	BD Biosciences	G46-6	5:100
CXCR4	PE	BD Biosciences	12G5	5:100
CD38	AF700	Biolegend	HIT2	3:100
CCR7	PE-Cy7	Biolegend	G043H7	2:100
CCR5	AF647	Biolegend	HEK/1/85a	3:100

Supplementary Table 5: Antibody panel for the analysis of HIV fusion in Tet+ CD4+ T cells

Marker	*fluorochrome*	*Manufacturer*	*Clone*	*Dilution*
TCR or Tetramer	APC	eBioscience/-	IP26 / -	1:100/-
Viability Dye	eFluor 780	eBioscience	-	1:200
CD4	PE-CF594	BD Biosciences	RPA-T4	1:200
CD45RA	BUV737	BD Biosciences	HI100	1:100
CD3	BUV395	BD Biosciences	SK7	5:100
CCF2-AM	Amcyan channel	Invitrogen	-	-
CCF2-AM-cleaved (Fused cells)	Pacific blue channel	Invitrogen	-	-
CCR7	PE-Cy7	Biolegend	G043H7	2:100
CCR5	AF700	Biolegend	HEK/1/85a	3:100

Supplementary Table 6: Antibody panel for the analysis of HIV fusion in total CD4+ T cells

Marker	*fluorochrome*	*Manufacturer*	*Clone*	*Dilution*
Viability Dye	eFluor 780	eBioscience	-	1:200
CD4	PE-CF594	BD Biosciences	RPA-T4	1:200
CD45RA	BUV737	BD Biosciences	HI100	1:100
CD3	BUV395	BD Biosciences	SK7	5:100
CD14	AF700	BD Biosciences	M5E2	3:100
CCF2-AM	Amcyan channel	Invitrogen	-	-
CCF2-AM-cleaved (Fused cells)	Pacific blue channel	Invitrogen	-	-
CCR7	PE-Cy7	Biolegend	G043H7	2:100
CCR5	AF647	Biolegend	HEK/1/85a	3:100

Supplementary Table 7: Antibody panel for the analysis of HIV fusion in CCR5-nucleofected CD4+ T cells

Marker	*fluorochrome*	*Manufacturer*	*Clone*	*Dilution*
Viability Dye	eFluor 780	eBioscience	-	1:200
CD4	BUV805	BD Biosciences	RPA-T4	1:200
CD45RA	BUV737	BD Biosciences	HI100	1:100
CD3	BUV395	BD Biosciences	SK7	5:100
CD14	AF700	BD Biosciences	M5E2	3:100
CCF2-AM	Amcyan channel	Invitrogen	-	-
CCF2-AM-cleaved (Fused cells)	Pacific blue channel	Invitrogen	-	-
CCR7	PE-Cy7	Biolegend	G043H7	2:100
CCR5 HEK/1/85a	AF647	Biolegend	HEK/1/85a	3:100
mCherry (reporter gene)				

Supplementary Table 8: Antibody panel for the analysis of CCR5 downregulation in patient CD4+ T cells

Marker	*fluorochrome*	*Manufacturer*	*Clone*	*Dilution*
CD3	eF780-APC	eBioscience	UCHT1	1:100
Viability Dye	eFluor 506	eBioscience	-	1:200
CD4	PE-CF594	BD Biosciences	RPA-T4	1:200
CD8	BV785	Biolegend	RPA-T8	1:200
CD45RA	BV421	Biolegend	HI100	1:100
CCR7	PE-Cy7	Biolegend	G043H7	2:100
CCR5	AF647	Biolegend	HEK/1/85a	3:100
CD69	FITC	BD Biosciences	FN50	1:100

Supplementary Table 9: Antibody panel for the analysis of CCR5 expression after antibody feeding

Marker	*fluorochrome*	*Manufacturer*	*Clone*	*Dilution*
Fixable Live/Dead Near-IR kit	APC-Cy7	Invitrogen	-	1:700
CD3	BV510	Biolegend	UCHT1	1:50
CD4	PE-CF-594	BD Biosciences	RPA-T4	1:200
CD8	BV785	Biolegend	RPA-T8	1:200
CD45RA	BV421	Biolegend	HI100	1:100
CD69	PE-Cy7	BD Biosciences	FN50	1:50
CD154	PE	BD Biosciences	TRAP1	1:20
CCR5 HEK/1/85a	AF647	Biolegend	HEK/1/85a	1:33

Supplementary Table 10: Antibody panel for the analysis of TCR-dependent regulation of CCR5 expression in TCR-transduced PBMC

Marker	*fluorochrome*	*Manufacturer*	*Clone*	*Dilution*
Viability Dye	eFluor 506	eBioscience	-	1:200
CD3	eF780-APC	eBioscience	UCHT1	1:100
CD4	PE-Cy7	BD Biosciences	SK3	1:100
CD8	BV785	Biolegend	RPA-T8	1:200
CD69	FITC	BD Biosciences	FN50	10:100
CCR5 HEK/1/85a	AF647	Biolegend	HEK/1/85a	3:100
mCherry (reporter gene)				

____
