# Supplementary material for: Low CCR5 expression protects HIV-specific CD4+ T cells of elite controllers from viral entry
Source: Nat Commun. 2022 Jan 26;13:521. doi: 10.1038/s41467-022-28130-0 (PMC8792008; doi:10.1038/s41467-022-28130-0)
Supplement: Supplementary file 2 — Reporting Summary [file 41467_2022_28130_MOESM2_ESM.pdf]

nature research

Corresponding author(s): L.A. Chakrabarti

Last updated by author(s): 21/12/2021

Reporting Summary

Nature Research wishes to improve the reproducibility of the work that we publish. This form provides structure for consistency and transparency in reporting. For further information on Nature Research policies, see our Editorial Policies and the Editorial Policy Checklist.

Statistics

For all statistical analyses, confirm that the following items are present in the figure legend, table legend, main text, or Methods section.

n/a	Confirmed	
☐	☒	The exact sample size (*n*) for each experimental group/condition, given as a discrete number and unit of measurement
☐	☒	A statement on whether measurements were taken from distinct samples or whether the same sample was measured repeatedly
☐	☒	The statistical test(s) used AND whether they are one- or two-sided<br>*Only common tests should be described solely by name; describe more complex techniques in the Methods section.*
☒	☐	A description of all covariates tested
☐	☒	A description of any assumptions or corrections, such as tests of normality and adjustment for multiple comparisons
☐	☒	A full description of the statistical parameters including central tendency (e.g. means) or other basic estimates (e.g. regression coefficient) AND variation (e.g. standard deviation) or associated estimates of uncertainty (e.g. confidence intervals)
☐	☒	For null hypothesis testing, the test statistic (e.g. *F*, *t*, *r*) with confidence intervals, effect sizes, degrees of freedom and *P* value noted<br>*Give P values as exact values whenever suitable.*
☒	☐	For Bayesian analysis, information on the choice of priors and Markov chain Monte Carlo settings
☒	☐	For hierarchical and complex designs, identification of the appropriate level for tests and full reporting of outcomes
☐	☒	Estimates of effect sizes (e.g. Cohen's *d*, Pearson's *r*), indicating how they were calculated

Our web collection on statistics for biologists contains articles on many of the points above.

Software and code

Policy information about availability of computer code

Data collection	- FACS data acquisition: FACSDiva software v8.0 (BD) , Attune NxT software v3.1 (ThermoFisher Scientific), and CytExpert Sofware v2.4 (Beckman Coulter)<br>- Single Cell multiplexed qPCR acquisition: Biomark Real time PCR analysis software v2.1 (Fluidigm)<br>- Long read CCR5 sequencing: SMRT Link v5.1 software on a Sequel instrument (Pacific Biosciences).
Data analysis	FACS data analysis: Flowjo v9 and v10 (BD).<br><br>Analysis of single cell gene and protein expression data:<br>- MAST (Model-based Analysis of Single cell Transcriptomics v1.0.5) package and linear modeling in the R (v3.3.3) software environment.<br>- Co-expression networks were inferred using the "huge" R package (v1.2.7).<br>- A Linear Discriminant Analysis (LDA) was performed on both MFI and genes using the MASS (v7.3-47) R package (Venables and Ripley, 2002).<br><br>Analysis of CCR5 sequences:<br>- CLC Genomics Workbench software v7.5 (Qiagen)<br>- Sequana v0.7.2 software (ref. 67)<br>- minimap2 aligner v2.8 software (ref. 68)<br>- freebayes v1.2 software (ref. 69)<br><br>Analysis of HIV tropism: The V3 region of HIV-1 Env was sequenced and tropism was determined by Geno2Pheno algorithm v2.5 (https://coreceptor.geno2pheno.org).

Other statistics were computed with the Prism v7.0 and v8.0 software (GraphPad).
No code was generated for this study.

For manuscripts utilizing custom algorithms or software that are central to the research but not yet described in published literature, software must be made available to editors and reviewers. We strongly encourage code deposition in a community repository (e.g. GitHub). See the Nature Research guidelines for submitting code & software for further information.

Data

Policy information about availability of data

All manuscripts must include a data availability statement. This statement should provide the following information, where applicable:

- Accession codes, unique identifiers, or web links for publicly available datasets
- A list of figures that have associated raw data
- A description of any restrictions on data availability

Source data are provided with this paper. Data corresponding to Figures 1 to 8 and Supplementary Figures 1 to 10 are provided in the accompanying Source Data excel file.
CCR5 sequences have been deposited to the European Nucleotide Archive (ENA) under the ArrayExpress accession code E-MTAB-11062 [https://www.ebi.ac.uk/arrayexpress/experiments/E-MTAB-11062/].

Field-specific reporting

Please select the one below that is the best fit for your research. If you are not sure, read the appropriate sections before making your selection.

[x] Life sciences [ ] Behavioural & social sciences [ ] Ecological, evolutionary & environmental sciences

For a reference copy of the document with all sections, see nature.com/documents/nr-reporting-summary-flat.pdf

Life sciences study design

All studies must disclose on these points even when the disclosure is negative.

Sample size	Given the exploratory nature of the study, we did not perform statistical analyses to predetermine sample size.
Data exclusions	Datapoints were exluded from the single cell analysis if the cell did not pass the initial quality control, defined by sufficient expression of the GADPH gene (Ct <18). Genes showing weak expression due to primer dimer accumulation, as visualized on PCR melting curves, were also removed from the analysis. During quality control associated to the statistical analysis, a total of 40 out of 740 sorted cells were considered as outliers and were removed from the single cell analysis.
Replication	Biological replicates represent different donors or patients. Experiments were performed on at least 2 biological replicates unless specified in the figure legend. All attempts at replication were successful.
Randomization	Randomization was not appropriate for this study as cells from each healthy donor or patient were used as their own control (Tetramer+ vs Tetramer- cells, unstimulated vs stimulated conditions, ...)
Blinding	Blinding of patient identity was performed by the methodological team of the CODEX cohort headed by Dr Faroudy Boufassa. Blinding regarding group assignments was not implemented in this study, since no subjective variables were measured.

Reporting for specific materials, systems and methods

We require information from authors about some types of materials, experimental systems and methods used in many studies. Here, indicate whether each material, system or method listed is relevant to your study. If you are not sure if a list item applies to your research, read the appropriate section before selecting a response.

Materials & experimental systems

n/a	Involved in the study	
[ ]	[x]	Antibodies
[ ]	[x]	Eukaryotic cell lines
[x]	[ ]	Palaeontology and archaeology
[x]	[ ]	Animals and other organisms
[ ]	[x]	Human research participants
[ ]	[x]	Clinical data
[x]	[ ]	Dual use research of concern

Methods

n/a	Involved in the study	
[x]	[ ]	ChIP-seq
[ ]	[x]	Flow cytometry
[x]	[ ]	MRI-based neuroimaging

Antibodies

Antibodies used

CD3, eF780-APC, eBioscience, clone UCHT1, ref. # 47-0038-42
TCR, APC, eBioscience, clone IP26, ref. # 17-9986-42
CD4, PE-CF594, BD Biosciences, clone RPA-T4, ref. 562281
CXCR5, AF488, BD Biosciences, clone RF8B2, ref. 558112
CXCR3, BV605, BD Biosciences, clone 1C6/CXCR3, ref. 564032
CD14, Viogreen, Miltenyi Biotec, clone TÜK4, ref. 130-113-715
CD20, Viogreen, Miltenyi Biotec, clone LT29, ref. 130-113-941
CD8, BV785, Biolegend, clone RPA-T8, ref. 301045
CD45RA, BV421, Biolegend, clone HI100, ref. 304129
CCR7, PE-Cy7, Biolegend, clone G043H7, ref. 353225
CCR5, PerCP-Cy5-5, Biolegend, clone HEK/1/85a, ref. 313715
CD3, BUV395, BD Biosciences, clone SK7, ref. 564001
HLA-DR, FITC, BD Biosciences, clone G46-6, ref. 556643
CXCR4, PE, BD Biosciences, clone 12G5, ref. 555974
CD38, AF700, Biolegend, clone HIT2, ref. 303523
CCR5, AF647, Biolegend, clone HEK/1/85a, ref. 313711
CD45RA, BUV737, BD Biosciences, clone HI100, ref. 612846
CCR5, AF700, Biolegend, clone HEK/1/85a, ref. 313713
CD14, AF700, BD Biosciences, clone M5E2, ref. 561029
CD4, BUV805, BD Biosciences, clone RPA-T4, ref. 742000
CD69, FITC, BD Biosciences, clone FN50, ref. 560969
CD3, BV510, Biolegend, clone UCHT1, ref. 300447
CD69, PE-Cy7, BD Biosciences, clone FN50, ref. 561928
CD154, PE, BD Biosciences, clone TRAP1, ref. 561720
CD4, PE-Cy7, BD Biosciences, clone SK3, ref. 557852
CCL3, goat polyclonal Ab, R&D Systems, ref. #AF-270-NA
CCL4, goat polyclonal Ab, R&D Systems, ref. #AF-271-NA
CCL5, goat polyclonal Ab, R&D Systems, ref. #AF-278-NA
Flag, Sigma Aldrich, clone M1, ref. F3040
Goat anti-mouse secondary Ab, PE, BD Biosciences, ref. 550589

Validation

All antibodies were validated by their manufacturer. Commercial antibodies chosen were further validated by citations in the primary literature. Relevant citations:

CD3, eF780-APC, eBioscience, clone UCHT1DOI: 10.1080/2162402X.2018.1505174
DOI: 10.1172/JCI99629
DOI: 10.1016/j.ccell.2018.08.017

TCR, APC, eBioscience, clone IP26
DOI: 10.1172/JCI125957
DOI: 10.3389/fimmu.2018.01062
DOI: 10.1038/s41419-018-1295-1

CD4, PE-CF594, BD Biosciences, clone RPA-T4
DOI: 10.15252/emmm.202013901
DOI: 10.1038/s41467-020-17489-7

CXCR5, AF488, BD Biosciences, clone RF8B2
DOI: 10.1042/bj3090773
DOI: 10.1002/eji.1830221107
DOI: 10.1038/35876

CXCR3, BV605, BD Biosciences, clone 1C6/CXCR3
DOI: 10.1084/jem.184.3.963
DOI: 10.1172/JCI1422

CD14, Viogreen, Miltenyi Biotec, clone TÜK4
DOI: 10.4049/jimmunol.165.11.6037
DOI: 10.1182/blood-2010-01-264218

CD20, Viogreen, Miltenyi Biotec, clone LT29
DOI: 10.1182/blood-2008-01-134783
DOI: 10.1634/stemcells.20-3-215

CD8, BV785, Biolegend, clone RPA-T8

DOI: 10.4049/jimmunol.1700953
DOI: 10.1016/j.celrep.2019.12.050

CD45RA, BV421, Biolegend, clone HI100DOI: 10.4049/jimmunol.1700953
DOI: 10.3389/fimmu.2018.00385

CCR7, PE-Cy7, Biolegend, clone G043H7
DOI: 10.4049/jimmunol.1700953
DOI: 10.1016/j.cell.2018.08.011

CCR5, PerCP-Cy5-5, Biolegend, clone HEK/1/85a
DOI: 10.1371/journal.pone.0042217
DOI: 10.4049/jimmunol.1302732

CD3, BUV395, BD Biosciences, clone SK7
DOI: 10.1038/s41591-020-1054-6
DOI: 10.3892/ol.2017.6294

HLA-DR, FITC, BD Biosciences, clone G46-6
DOI: 10.1084/jem.193.11.1303
DOI: 10.4049/jimmunol.175.5.3431

CXCR4, PE, BD Biosciences, clone 12G5
DOI: 10.1016/s0092-8674(00)81393-8
DOI: 10.1126/science.272.5263.872

CD38, AF700, Biolegend, clone HIT2
DOI: 10.1016/j.celrep.2019.08.037
DOI: 10.1128/mBio.00317-18

CCR5, AF647, Biolegend, clone HEK/1/85a
DOI: 10.1016/j.immuni.2021.05.010
DOI: 10.1182/blood-2011-08-372516

CD45RA, BUV737, BD Biosciences, clone HI100
DOI: 10.4049/jimmunol.1101760
DOI: 10.1016/j.cell.2020.11.029

CCR5, AF700, Biolegend, clone HEK/1/85a
DOI: 10.1073/pnas.1400446111
DOI: 10.4049/jimmunol.1302732

CD14, AF700, BD Biosciences, clone M5E2
DOI: 10.1126/science.1698311

CD69, FITC, BD Biosciences, clone FN50
DOI: 10.1002/(SICI)1097-0320(19960701)24:3<191::AID-CYTO1>3.0.CO;2-L

CD3, BV510, Biolegend, clone UCHT1
DOI: 10.1084/jem.20171940
DOI: 10.1038/ncomms12624

CD69, PE-Cy7, BD Biosciences, clone FN50
DOI: 10.1002/(SICI)1097-0320(19960701)24:3<191::AID-CYTO1>3.0.CO;2-L

CD154, PE, BD Biosciences, clone TRAP1
DOI: 10.1172/JCI117089

CD4, PE-Cy7, BD Biosciences, clone SK3
DOI: 10.1002/(SICI)1097-0320(19960701)24:3<191::AID-CYTO1>3.0.CO;2-L

CD4, BUV805, BD Biosciences, clone RPA-T4
DOI: 10.1016/j.ebiom.2021.103241

CCL3, goat polyclonal Ab, R&D Systems, ref. #AF-270-NA
DOI: 10.1016/j.celrep.2019.08.050
DOI: 10.1128/JVI.00118-15

CCL4, goat polyclonal Ab, R&D Systems, ref. #AF-271-NA
DOI: 10.1128/JVI.00118-15
DOI: 10.4049/jimmunol.1400417

CCL5, goat polyclonal Ab, R&D Systems, ref. #AF-278-NA
DOI: 10.1038/s41467-021-24386-0
DOI: 10.1038/s41598-018-19643-0

Flag, Sigma Aldrich, clone M1, ref. F3040
DOI: 10.1371/journal.pone.0083114

Eukaryotic cell lines

Policy information about cell lines

Cell line source(s)	HEK 293T cells were obtained from the ECACC.
Authentication	The HEK 293T cells were not authenticated.
Mycoplasma contamination	HEK 293T cells were routinely tested for mycoplasma contamination and were found negative.
Commonly misidentified lines (See ICLAC register)	No commonly misidentified cell lines were used in this study.

Human research participants

Policy information about studies involving human research participants

Population characteristics	HIV controllers were defined as HIV-1-infected patients who had been seropositive for >5 years, who had received no antiretroviral treatment, and for whom >90% of plasma viral load measurements were undetectable by standard assays. All HIV controllers included in the study had viral loads of <50 copies/ml at inclusion.<br>Efficiently treated patients had received antiretroviral therapy for a minimum of 5 years and showed long-term HIV-1 suppression with viral loads of <50 copies/ml.<br>The duration of infection, viral load, CD4+ T cell count, nadir of CD4+ T cells, and age are compared for the two patient groups in Supplementary Table 1A.
Recruitment	HIV controllers (HIC group; n=25) were recruited through the CO21 CODEX cohort set up by the Agence Nationale de Recherche sur le SIDA et les Hépatites Virales (ANRS).<br>Treated patients (ART group; n=24) were recruited at the Raymond Poincaré and Bicêtre hospitals (France).<br>Healthy donors were anonymous volunteers who donated blood at the Etablissement Français du Sang.<br>Potential self-selection and recruiting biases are unlikely to affect the parameters measured in this study.
Ethics oversight	The study was promoted by ANRS and approved by the Comité de Protection des Personnes IDF-VII under number 11-33. All participants gave written informed consent prior to inclusion in the study.

Note that full information on the approval of the study protocol must also be provided in the manuscript.

Clinical data

Policy information about clinical studies
All manuscripts should comply with the ICMJEguidelines for publication of clinical research and a completedCONSORT checklist must be included with all submissions.

Clinical trial registration	The present study is not based on a clinical trial. The study is based on ancillary studies EP36-10 , EP36-11, and EP36-13 to the ANRS CO21 CODEX cohort. The cohort focuses on the study of HIV-infected patients with an "extreme" profile, including HIV controllers.
Study protocol	The full protocol of the CODEX cohort can be obtained from ANRS (www.anrs.fr).<br>A summarized description of the cohort can be found at:<br>https://www.anrs.fr/sites/default/files/2020-11/Fiche%20web_ANRS%20CO21%20CODEX.pdf
Data collection	Samples were collected from hospitals declared as ANRS Centers in the Ile de France Region, from Dec. 2014 to July 2021.
Outcomes	The present study was observational. No clinical outcomes were monitored.

Flow Cytometry

Plots

Confirm that:

- [x] The axis labels state the marker and fluorochrome used (e.g. CD4-FITC).
- [x] The axis scales are clearly visible. Include numbers along axes only for bottom left plot of group (a 'group' is an analysis of identical markers).
- [x] All plots are contour plots with outliers or pseudocolor plots.
- [x] A numerical value for number of cells or percentage (with statistics) is provided.

Methodology

Sample preparation	Peripheral blood mononuclear cells (PBMC) were isolated from heparinized blood via density gradient centrifugation on Ficoll-Paque PLUS (GE Healthcare Life Sciences) and were either cryopreserved or used freshly for the preparation of monocyte-derived dendritic cells (MDDC). After differentiation in GM-CSF and IL-4, MDDC were collected and cryopreserved until use in antigen presentation experiments. All experiments were done on thawed PBMC and MDDC.
Instrument	The flow cytometry analyzers used were a FACS BD Fortessa, a BD Symphony, a ThermoFischer Attune NxT , and a Beckmazn Coulter Cytoflex apparatus. The cell sorter used was a BD FACSAria III apparatus.
Software	Acquisitions on the BD Fortessa, Symphony, and FASCAria III machines were perfomed with the FACSDiva software (BD).<br>Acquisitions on the Attune NxT cytometer were performed with the Attune NxT software (ThermoFisher Scientific).<br>Acquisitions on the Cytoflex cytometer were performed with the CytExpert software (Beckman Coulter).<br>All analyses were performed with the Flowjo v9 and v10 softwares (BD).
Cell population abundance	For the single-cell sorting of tetramer+ (Tet+) and tetramer- (Tet-) CR45RA- CD4+ T cells, 25 Tet + and 25 Tet- cells were sorted for each patient studied, using the single cell purity mode to avoid doublets. The "index sorting" mode was used to record the individual fluorescence parameters of each sorted cell.
Gating strategy	Doublets and cell debris were removed by FSC-H/FSC-A and SSC-H/SSC-W gating, followed by FSC-A/SSC-A morphology gating. Dead cells were removed by viability staining-. The detailed list of antibody panels used is provided in the Methods section.

- [x] Tick this box to confirm that a figure exemplifying the gating strategy is provided in the Supplementary Information.
